# Investigation of sequence features of hinge-bending regions in proteins with domain movements using kernel logistic regression

**DOI:** 10.1186/s12859-020-3464-3

**Published:** 2020-04-09

**Authors:** Ruth Veevers, Gavin Cawley, Steven Hayward

**Affiliations:** Computational Biology Laboratory, School of Computing Sciences, University of East Anglia, Norwich, NR4 7TJ UK

**Keywords:** Protein conformational change, Domain closure, Hinge axis, Linker region

## Abstract

**Background:**

Hinge-bending movements in proteins comprising two or more domains form a large class of functional movements. Hinge-bending regions demarcate protein domains and collectively control the domain movement. Consequently, the ability to recognise sequence features of hinge-bending regions and to be able to predict them from sequence alone would benefit various areas of protein research. For example, an understanding of how the sequence features of these regions relate to dynamic properties in multi-domain proteins would aid in the rational design of linkers in therapeutic fusion proteins.

**Results:**

The DynDom database of protein domain movements comprises sequences annotated to indicate whether the amino acid residue is located within a hinge-bending region or within an intradomain region. Using statistical methods and Kernel Logistic Regression (KLR) models, this data was used to determine sequence features that favour or disfavour hinge-bending regions. This is a difficult classification problem as the number of negative cases (intradomain residues) is much larger than the number of positive cases (hinge residues). The statistical methods and the KLR models both show that cysteine has the lowest propensity for hinge-bending regions and proline has the highest, even though it is the most rigid amino acid. As hinge-bending regions have been previously shown to occur frequently at the terminal regions of the secondary structures, the propensity for proline at these regions is likely due to its tendency to break secondary structures. The KLR models also indicate that isoleucine may act as a domain-capping residue. We have found that a quadratic KLR model outperforms a linear KLR model and that improvement in performance occurs up to very long window lengths (eighty residues) indicating long-range correlations.

**Conclusion:**

In contrast to the only other approach that focused solely on interdomain hinge-bending regions, the method provides a modest and statistically significant improvement over a random classifier. An explanation of the KLR results is that in the prediction of hinge-bending regions a long-range correlation is at play between a small number amino acids that either favour or disfavour hinge-bending regions. The resulting sequence-based prediction tool, HingeSeek, is available to run through a webserver at hingeseek.cmp.uea.ac.uk.

## Background

Protein domains have various definitions within Biochemistry [1]. From a structural perspective a domain is characterised as a globular, spatially separate part of a protein and methods have been developed to recognise them from this property [2]. They are considered to be able to fold independently of other parts of the protein and are associated with a distinct function. This lends them the ability to act as a fundamental component of evolutionary change. For protein structure databases such as SCOP [3], SCOP2 [4] and CATH [5] they form the basic element of classification. They can be identified from sequence homology using methods such as Pfam [6] where multiple-sequence alignments of family members of a domain are encoded as hidden Markov models.

It is now an established fact that conformational change is integral to protein function [7, 8]. A common class of movement is a domain movement in proteins comprising more than one domain [9–12]. Several methods have been developed to identify domains from the movement itself [13–18] and in this context they have been called “dynamic domains”. The relative movement of dynamic domains is controlled by so-called hinge-bending regions located between the domains. These normally comparatively short regions collectively control the domain movement [10] as has been demonstrated using inverse-kinematics Monte Carlo in glutamine binding protein where the known domain movement was reproduced almost perfectly when only 11 of the 226 residues situated at the two hinge-bending regions were allowed to flex [19]. In an early application of the DynDom method it was found that hinge-bending regions are often situated at the termini of β-sheets and α-helices [10].

To date very little work has been carried out to determine whether hinge-site features are reflected in the sequence. Flores et al. [20] annotated hinge-bending regions from the Database of Macromolecular Motion (DBMM) [21] to form their “Hinge Atlas” dataset and performed statistical analyses to create a predictor for hinge sites from sequence alone. Hinge sites were identified using the FlexProt program [22]. They calculated log-odds frequencies scores for a 17-residue-long sliding window, assigning the central residue to a hinge-bending region if the resulting accumulated score was above a threshold. The results achieved did not appear to be significantly different to a random assignment. They incorporated information about secondary structure and active site location into the predictor, “HingeSeq”, which improved predictive power. They did not quote the area under the ROC curve (AUROC) but we estimated it from their figure to be approximately 0.65.

Kuznetsov [23] reports using support vector machines (SVM) to predict “conformational switches” from sequence, which were described as areas of flexibility that drive conformational change. The basic data used also came from the DBMM but the sites identified, based on changes in main-chain dihedral angles, were not exclusively located at hinge-bending regions. Using a window length of 11 residues, an AUROC of 0.64 was found, which increased to 0.69 when profiles were used. The method has been implemented at the webserver FlexPred [24]. Bodén and Bailey [25] presented a method, also based on the DBMM, which predicted “conformational variability” based on secondary structure prediction uncertainty for which a neural network was used. A window length of 15 was used and an AUROC of 0.64 was reported.

This work relates also to the study of linker regions; polypeptide regions that link two domains [26, 27]. The difference between these linker region studies and hinge-bending region/conformational-switch region studies, is that the latter were identified from conformational change, whereas the former were identified purely on structural features. There is an increasing interest in the dynamic properties of linker regions as their rational design would benefit the efficacy of therapeutic fusion proteins constructed using recombinant DNA technology [28].

**Fig. 1:**
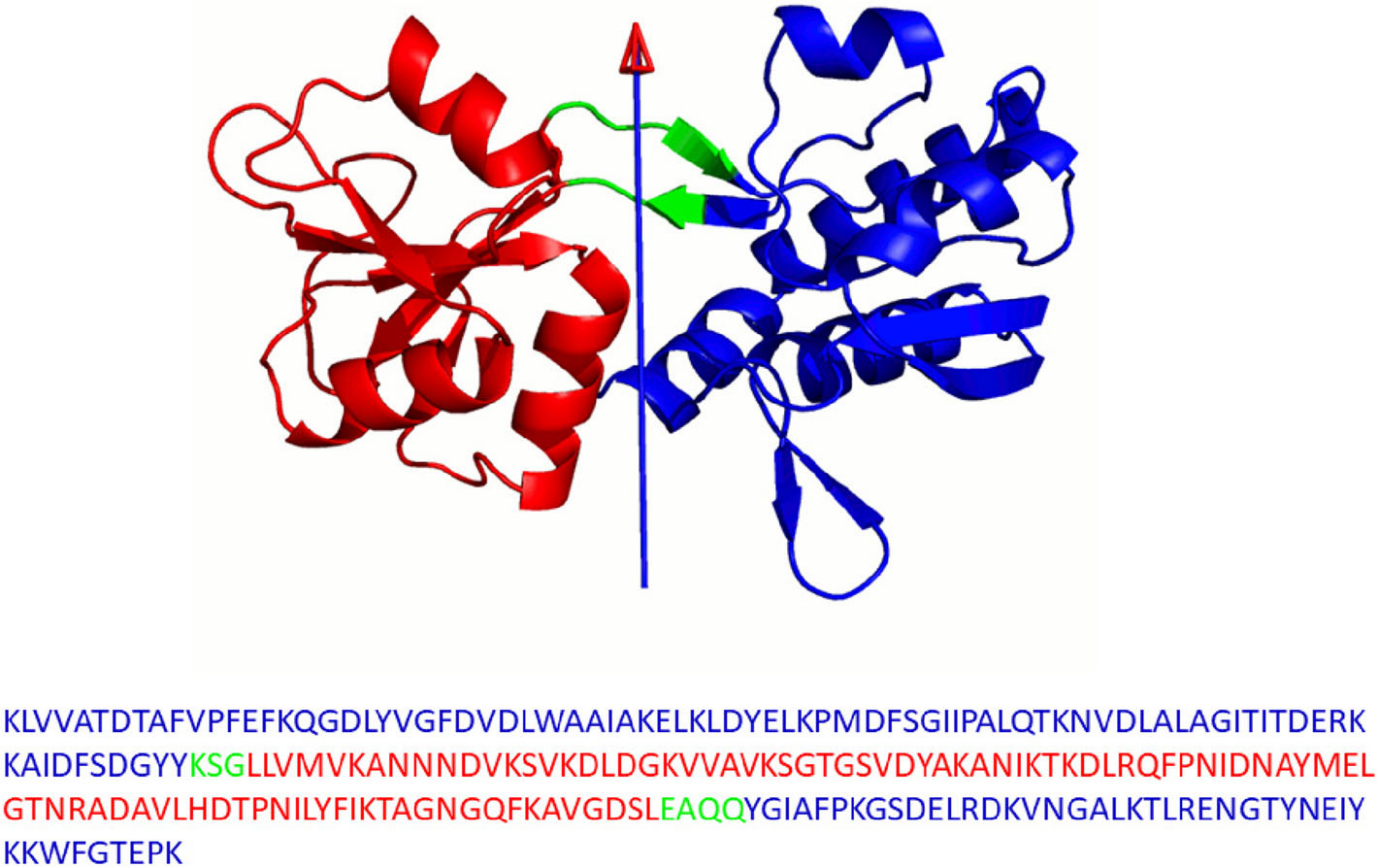
DynDom result for glutamine binding protein. DynDom result for the movement that occurs upon binding glutamine (PDB: 1GGG, chain A to PDB: 1WDN, chain A) showing the open, ligand-free conformation (see DynDom website at www.cmp.uea.ac.uk/dyndom for more details on this and other domain movements). The arrow represents the hinge axis. Red and blue are the dynamic domains, green the hinge-bending regions. Red and blue amino acids in the sequence at the bottom of the figure are intradomain and green amino acids are hinge-bending. Such annotated sequences are the basic data of this study. This is a typical member of Group 1 (see Methods)

A feature of the DynDom program is that it determines not only dynamic domains but also hinge-bending regions, as can be seen in the example of glutamine binding protein in Fig. 1. Dynamic domains are determined based on their rotational properties and hinge-bending regions are those regions within which a rotational transition occurs in going from one dynamic domain to another. This connects directly with what “bending” really means. The exact method for assigning bending regions is described in detail by Hayward and Lee [29]. This precise definition of a bending region lends itself to the aim of this study. Here we trained a range of Kernel Logistic Regression (KLR) models on protein sequences with hinge-site annotation from examples that showed a clear hinge-bending movement in the two main DynDom databases in order to understand sequence properties of hinge-bending regions and to produce a hinge site predictor from sequence.

## Results

### Hinge statistics

**Fig. 2:**
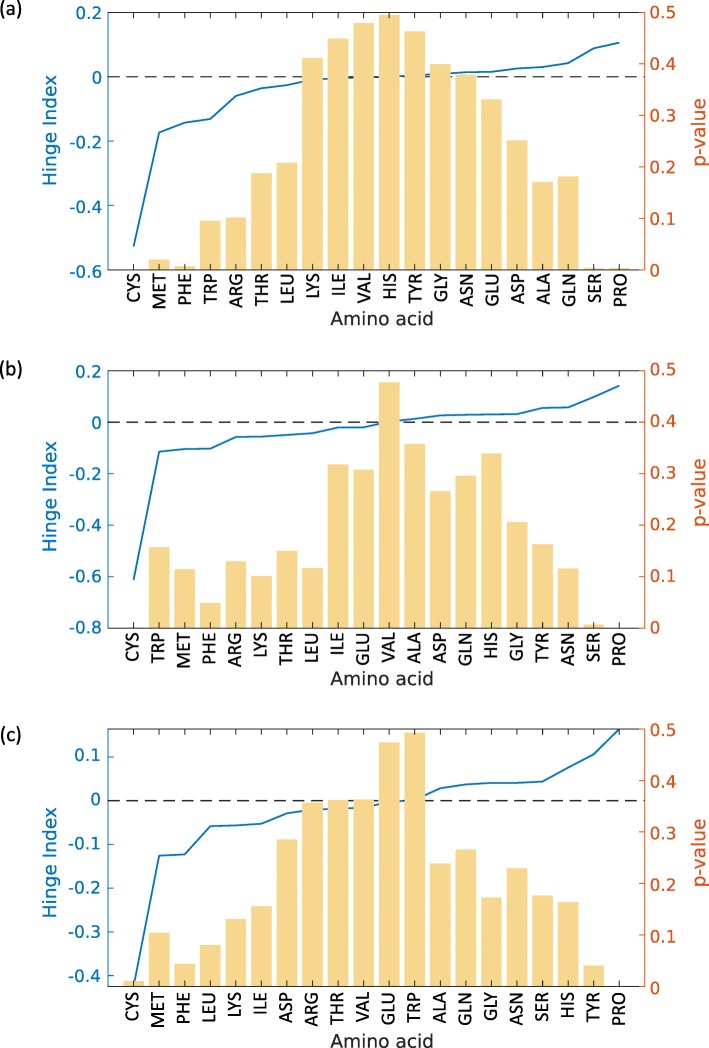
Propensities (Hinge Index, *HI*) of amino acids and *p*-values. The *HI* and p-value of each amino acid for the following datasets (the percentage sets the filtering level according to sequence identity; see Methods section for definitions): **a** Group1_90% **b** Group1_40% **c** Group1_20%. The amino acids have been sorted according to their *HI* values (blue lines). A negative *HI* value indicates an amino acid that disfavours hinge-bending regions and a positive value indicates an amino acid that favours them. The horizontal black broken line at *HI* = 0 indicates those with no preference. The light-brown bars indicate the *p* values

The Hinge Index, *HI*(*a*), for each amino acid, *a*, is shown in Fig. 2 for all three Group 1 datasets, that is Group1_90%, Group1_40% and Group1_20%. A negative *HI*(*a*) would indicate an amino acid that is unfavourable to hinge regions, a value of zero, an amino acid that has no preference, and a positive value an amino acid favourable to hinge regions. Although the results are generally supportive of those found by Flores et al., they are statistically significant only for a few amino acids in both studies. For Flores et al. Ser and Gly had the highest significant *HI* values. Here, Pro has the highest significant *HI* value at all three levels of filtering. We also found Ser to have a high significant *HI* value at 90 and 40% filtering, but contrary to expectation, Gly was not in the top four at any level of filtering.

At all levels of filtering, Cys received the most negative significant *HI* value and by a large margin. Phe and Met also disfavour hinge regions, Phe being the amino acid with the most negative *HI* value for Flores et al.. The β-branched amino acids Ile, Val and Thr all seem to weakly disfavour hinge regions although the results are not statistically significant.

The equivalent analysis on the Group2_90% is shown in Additional Figure 1. The results broadly agree with the Group1_90% results.

### KLR on 90% sequence identity set

#### Group 1

**Fig. 3:**
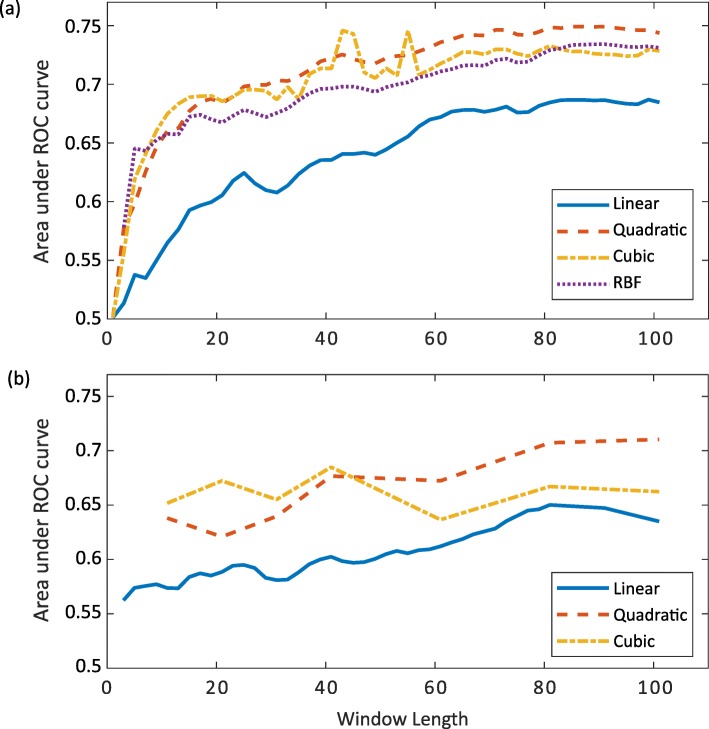
The performance of KLR models. Results show differences between the linear, quadratic, cubic and RBF models trained across a range of window lengths. **a** Group1_90% **b** Group2_90%

We trained KLR models with linear, quadratic, cubic, and RBF kernels on the training subset from Group1_90% (see Table 1). Each KLR model was constructed across a range of window lengths, *w* = [1, 101], and tested on the test set comprising 10% of the whole set selected at random. ROC curves were created for each window length and each kernel, plotting the rate of true positive outcomes against the rate of false positive outcomes. The AUROCs were calculated, giving a measure of performance for each combination of window length and kernel, as a number between zero and one, where higher numbers represent better performance. Figure 3a shows how these AUROCs change across window lengths for each kernel in Group1_90%. A classifier with an AUROC of 0.5 would be equivalent to assigning samples to the “hinge-bending region” or “not hinge-bending region” classes at random. There are two main things to notice about these results. First is that there is improvement in AUROC up until very long window lengths. This result is in contrast to previous studies on hinge-bending/conformationally-variable regions where windows of length less than 25 residues were used by Kuznetsov [23], a window of 17 residues by Flores et al. [20], and a window of 15 residues by Bodén and Bailey [25]. Here we see an improvement in AUROC with window lengths up to 80–90 residues. This suggests that if the window spans from one hinge-bending region to the next it can help prediction. The other noticeable feature is that the quadratic, cubic, and RBF kernels all seem to outperform the linear approach. Additional Table 1 shows a matrix of *p*-values for the pairwise comparisons of the AUROC for the four different models for window length 99 residues using Sun and Xu’s implementation [30] of the method by DeLong et al. [31]. The DeLong et al. method tests the null hypothesis that the difference in the empirical AUROCs can be adequately explained by the variance of the estimator. The null hypothesis is rejected when *p* < 0.05. This shows that all non-linear models significantly outperform the linear model, but that the non-linear models do not all significantly outperform each other. That the cubic model and RBF models do not improve performance over the quadratic model suggests that the quadratic terms are mainly where the improvement lies. This implies that there exists a correlation between certain pairs of residues at different positions within the window. The maximum value for the AUROC of 0.75 occurred for the quadratic model with a window length of 87 residues. The maximum value of the AUROC for the linear model was 0.69 with a window length of 99 residues.

**Table 1:** Selection criteria for Groups 1 and 2 and number of examples

Criterion	Group 1	Group 2
N^o^ of domains	2	2
Min n^o^ of residues in domain	80	80
Min angle of rotation	20°	15°
Max intradomain backbone RMSD	2.5 Å	3.0 Å
Max n^o^ of bending regions	3	5
Max n^o^ of residues in a bending region	10	15
Number of domain movements before CD-Hit filtering (90%)	910	1389
Number of domain movements after CD-Hit filtering (90%)^a^	241	372
Number of domain movements after CD Hit filtering (40%)	171	268
Number of domain movements after CD-Hit filtering (20%)	136	222

^a^See Additional Data_1 for list of pairs of structures by protein name and PDB codes

As stated in the Methods section, the ratio of positive to negative cases was adjusted to 1:9 for the training set, but in the test set the proportion of residues that are in hinge regions is only 0.0294 indicating a large class imbalance. In Additional Figure 2(A) we show a set of ROC curves and their AUROCs from the quadratic model with a window length 81 that uses different proportions of positive to negative cases in the training sets. We also show in Additional Figure 2(B), plots of how the AUROC varies with this proportion for different window lengths. These results confirm that KLR is reasonably robust to class imbalance as there is little change in the AUROCs with change in this proportion.

In Additional Figure 3 we show the Precision-Recall plot for window length 81. Such a plot emphasises the classification of positive examples. The area under the Precision-Recall plot (AUPRC), which is dependent on the class imbalance ratio, is 0.1785. A random classifier would give an AUPRC of 0.0294, the proportion of hinge residues in the test set. Additional Figure 4 shows the AUPRCs plotted against window length for the four different KLR models. The result mirrors the equivalent plot for the AUROCs.

#### Group 2

The Group2_90% was used for the same set of experiments as Group1_90%, although due to the greatly increased computational expense resulting from the use of this larger training set, fewer window lengths were tried although they spanned the same range (Fig. 3b). Again we found the same increase in performance with window length and the same improvement of the non-linear models over the linear model. The matrix of *p*-values in Additional Table 2 determined with DeLong et al.’s method, shows that the difference between the non-linear models and the linear model was statistically significant. In comparison with Group1_90%, each model performed worse at most window lengths indicating the negative influence of the less strict selection criteria for Group2_90%.

### KLR on 40% sequence identity set

**Fig. 4:**
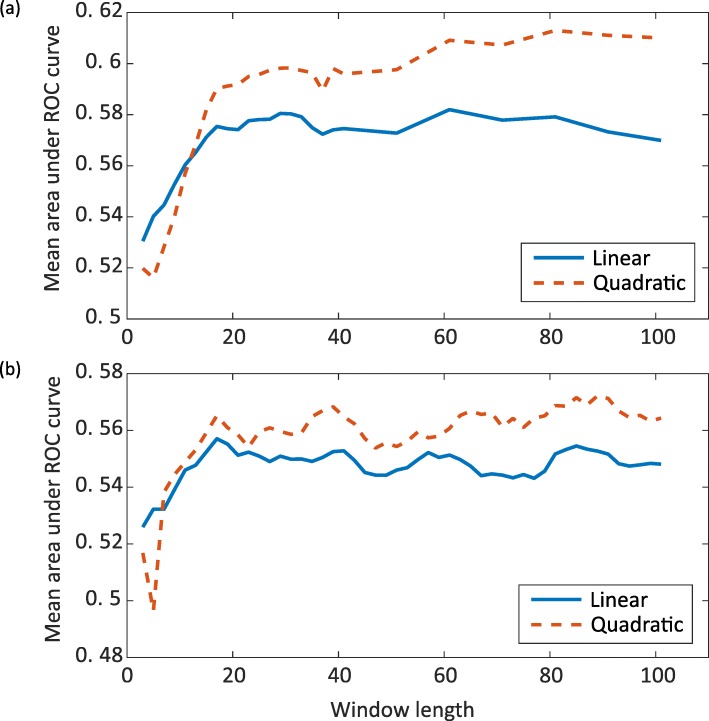
The mean AUROCs for linear and quadratic kernels. **a** Group1_40%. **b** Group1_20%

We considered whether the 90% sequence identity might permit similar sequences to be present in both training and test sets. The Group1 dataset contains 48 chains from immunoglobulins; pairwise comparisons between these sequences resulting in sequence identities ranging between 19.2 and 88.9%. We repeated the experiment for linear and quadratic models on the Group1_40% dataset, within which pairs of structures are less likely to be homologous [32]. This reduced the number of immunoglobulins included to 3 of 171 proteins. As this reduced the size of the dataset (see Table 1), we performed 10-fold cross validation (nested cross-validation was used in order to obtain an unbiased performance estimate [33]). Figure 4a shows the mean AUROC of the folds across windows of length 3 to 41 in increments of 2, and 41 to 101 in increments of 10. The results for both linear and quadratic kernels were poorer than the Group1_90% results, which is expected as there is less data in the training set. The models both improved at longer window lengths: the mean AUROC for the quadratic kernel was 0.61 achieved at window length 81, and the linear kernel peaked at a mean AUROC of 0.57 at 61 residues. *p*-values for paired t-tests across the folds for different window lengths is shown in Additional Figure 5. Additional Figure 5 shows that the longer the window, the lower the p-value becomes for the difference between the quadratic and linear model. At a window length 81 the p-value is 0.004 indicating a statistically significant improvement of the quadratic model over the linear model at long window lengths. Across the folds the AUPRC has a value mean value of 0.0415 compared to a mean ratio of hinge residues to all residues of 0.0232.

### KLR on 20% sequence identity set

We repeated these experiments using the Group1_20% dataset. As our original dataset is relatively small, filtering at the 20% level reduces the amount of data to an even lower level (see Table 1). Again we performed 10-fold cross validation. Figure 4b shows the mean AUROC of the folds across the same range of window lengths used for 40 and 90% filtering. As expected the results for both linear and quadratic kernels were poorer than the 90 and 40% results. Although the difference between the linear and quadratic models was not found to be significant using the paired t-test (which is likely due to the small amount of data), we do see the same trend as seen for the 90 and 40% results; that is an improvement in the AUROC of the quadratic model over the linear model at longer window lengths.

Across the folds the AUPRC has a value mean value of 0.0390 compared to a mean ratio of hinge residues to all residues of 0.0213.

### Analysis of model weights

In this section, we analyse the weights from the quadratic and linear kernels, at their optimal window lengths: 87 for Group1_90%, 81 for Group1_40%, and 87 for Group1_20%. The primal weight vector can be computed for finite feature spaces such as that of the linear and quadratic kernels, using Eq. 8.

#### Linear terms

**Fig. 5:**
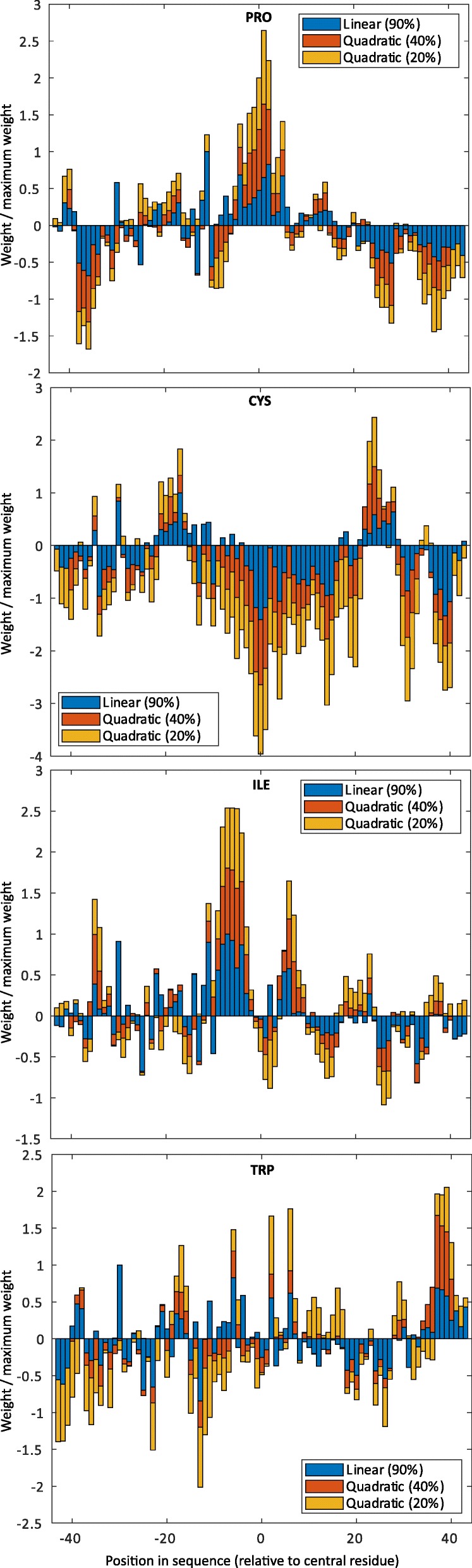
The linear weights assigned to Pro, Cys, Ile, and Trp. From top to bottom: Pro, Cys, Ile, and Trp by the linear KLR model at 90% filtering, and from the quadratic KLR models at 40 and 20% filtering. Window lengths were 87 for those trained using Group1_90%, 81 for those trained using Group1_40%, and 87 for those trained using Group1_20%

Figure 5 shows example plots of the linear weight distribution for given amino acids across the window. The scale of the weights differed between the linear and quadratic models, so each weight is represented as a proportion of the strongest weight applied by the model to the amino acid.

While there is some disagreement between the models, strong peaks and troughs can be observed at the same window positions for all three models. Pro was associated with strong positive weights in and around the central position, with negative weights ±40 residues from the central position. Pro has the highest positive weight of any amino acid at the central window position confirming the Hinge Index result. The weights in the Cys plots are mostly negative. It has the lowest valued weights at the central window position out of all amino acids. Interestingly it has pronounced positive weights around ±20 residues from the central position. The weights in the Ile plot fluctuate but all three models show strong positive weights around 5 residues on the N-terminal side of the central position and a smaller peak 5 residues after. These charts are not all approximately symmetrical; the Trp plot shows a strong positive peak around the end of the window, with no corresponding peak at the start.

#### Product terms

The feature space for the quadratic kernel includes features corresponding to the pairwise products of the original input attributes. The weights associated with product terms in the feature vectors give an indication of the strength of the importance of pairs of residues at different positions within the sliding window. These can be visualised for each amino acid pair by plotting them as a heat map, where each axis represents a position within the sliding window at which a residue occurs.

**Fig. 6:**
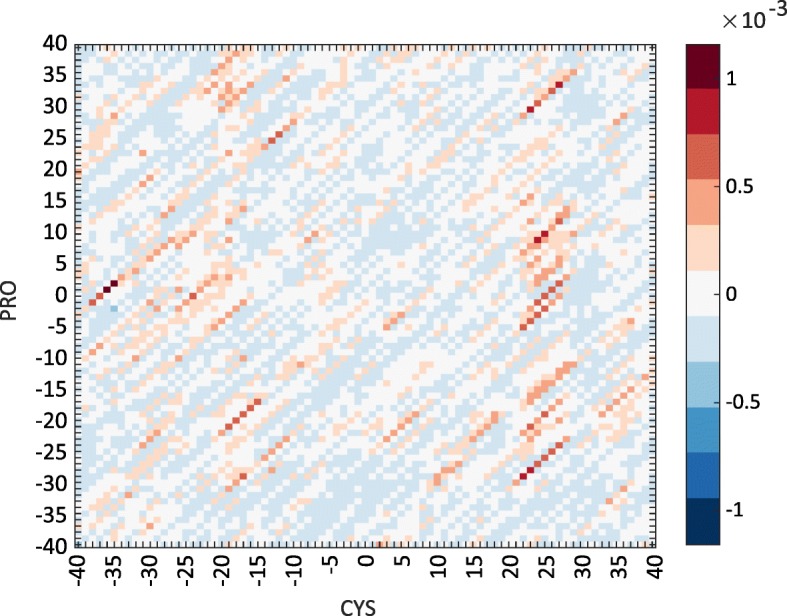
The weights assigned to combinations of Cys and Pro. Product term weights from quadratic kernel models with window length 81 trained using Group1_40%

The heat map in Fig. 6 shows the weights associated with combinations of Cys and Pro residues according to the quadratic model trained for the Group1_40% dataset. A patch of positive weights at position (20–25, 0–10) may indicate that such a combination is favoured. Structurally this would suggest a pair of domains with Pro located at a hinge-bending region and Cys located at an intradomain region on the C-terminal side. At this current time we cannot rule out the possibility that these correlations are an artefact of the small sample we have of non-homologous proteins with clear domain movements.

As optimal AUROCs predominantly occurred at window lengths of either 81 or 87, we include in Additional Table 3, AUROCs at both these window lengths (although AUROCs are not available for window length 87 on Group1_40% as we did not perform computations at this window length). The results show there is little or no difference between the AUROCs at these two window lengths.

### HingeSeek web server

We have produced a tool, called “HingeSeek”, which is available to run from a web server at hingeseek.cmp.uea.ac.uk. The server offers sequence-only hinge predictions, converting input sequences into windowed one-of-n encoded feature vectors and classifying each residue as hinge or non-hinge based on a selected threshold. The sequence is then coloured according to the classification, and labelled with the confidence level.

HingeSeek was created by bootstrapping the training data from Group1_90%. One hundred models were trained using the quadratic KLR model with the optimal window length of 87. Data was sampled with replacement creating training sets the same size as the original Group1_90% set. To allow unbiased assessment of the model’s predictions, there is a sequence identity threshold parameter. When a sequence is entered by the user, an ensemble is created such that no members of the ensemble were trained on any sequences having a greater sequence identity than the threshold with the input sequence. The weights are extracted from the selected models and averaged to create an aggregated model. This enables the tool to be used as a fair benchmark for comparison with competing approaches. In addition to allowing users to predict hinge-bending regions, the web server also includes an interactive weight explorer, which allows users to investigate the weights that the model assigned to amino acid pairings, by dynamically generating charts like Fig. 6.

## Discussion

We trained a range of KLR models on sequences taken from the DynDom database in order to understand sequence features of hinge-bending regions and to predict their locations from sequence alone.

With Group1_90%, a maximum AUROC of 0.75 was achieved. This contrasts favourably with Flores et al. [20] who could not achieve any predictive value using just Hinge Index information using the DBMM dataset also filtered at 90% sequence identity. With Group 1_40% and Group1_20%, the AUROC of the best KLR model was lower than the AUROC for the best KLR model using Group1_90%, probably due to the small amount of data available at these levels of sequence identity.

Beyond producing a sequence-based predictor for hinge regions, this work provides insight into what kinds of residue favour or disfavour hinge regions and hints at possible relationships between them. Broadly the residues found to favour hinge sites are those with small side chains confirming the finding by Flores et al. [20]. Ser strongly favours a hinge site even more so than Gly which, in contrast to Flores et al., we find only weakly favours hinge regions. Both for Group 1 and Group2, the Hinge Index analysis shows that Pro is the most favourable residue to be located at a hinge region and Cys the least favourable. This result is supported by an analysis of the weights of the linear-terms in the KLR models. The fact that Pro favours hinge-bending regions is unexpected as in contrast to all other amino acids rotation about its ϕ dihedral is severely restricted which one would think would inhibit its ability to act as a hinge-bending residue. This result concurs with studies on linker regions [26, 27] identified on structural features only. Such regions were intentionally omitted from our datasets as positive cases in order to be certain that those included were confined to those that demonstrably facilitate hinge bending. We believe the reason for Pro being located in these regions is that it often acts as a terminator for secondary structure elements and therefore appears at hinge regions because they are also often located at the terminal regions of secondary structures [10]. Cys is highly disfavoured at bending regions which can be explained by the fact that many Cys residues form disulphide bonds helping to rigidify the local backbone. Positive weights for Cys at the ± ~ 20 positions probably indicate the role it plays in stabilising a domain via cross-linking. Interestingly Ile appears to act as a domain-capping residue. The preference of some residues to be situated in bending regions and the preference of others for being located within a globular domain may explain why we see improvement in prediction up to comparatively long window lengths.

The consistently better performance of the quadratic kernel over the linear kernel at very long window lengths implies a correlation between amino acid locations which we believe occurs between a small number of amino acids, such as Pro and Cys, that particularly favour or disfavour hinge bending regions.

## Conclusions

We have used statistical methods and machine learning methods to investigate sequence features of hinge-bending regions. This work presents an example of an attempt to analyse sequence features involved in the structure-dynamic relationship. There is an increased interest in these regions particularly in their role as linkers in therapeutic fusion proteins. First, we revisited the Hinge Index measure introduced by Flores et al. [20]. The results broadly confirm their findings for the propensities of particular amino acids to occur in hinge-bending regions. However, there are some differences, most notably the finding that proline is the amino acid that has the highest propensity to occur in a hinge-bending region. This is thought to be due to its secondary-structure breaking tendency as it is at the termini of secondary structures that hinge bending often occurs. Flores et al. found that the Hinge Index alone could not be used to produce a reliable predictor and so here we have used KLR. Although we have produced a tool with useful predictive power it has not achieved the same level of predictive power as when machine learning methods are applied to secondary structure prediction from sequence [34]. This problem represents a case where there is a large class imbalance with the number of intradomain residues vastly outweighing the number of hinge-bending residues. This means that with a limited amount of data, and as our results indicated, only a few of the 20 amino acids having expressed any strong preference for or aversion of hinge regions, the number of false positives is likely to be high. Using KLR models of increasing complexity we have found an interesting and quite unusual feature for the prediction of hinge-bending regions, namely that the quadratic model outperforms the linear model particularly at very long window lengths (in comparison to other methods that have been applied to the prediction of hinge-bending/conformationally-variable regions). This result points to prediction performance being enhanced by the correlation between those residues that strongly favour or disfavour hinge-bending regions at a considerable distances apart along the chain. Understanding the role that particular amino acids play in the formation of hinge regions will be of interest to those who practise protein engineering, particularly those who design linker regions in therapeutic fusion proteins.

## Methods

### Dataset

The primary data comprised 5248 domain movements from unique pairs of structures analysed by the DynDom program. These are deposited in both the user-created database [35] and the non-redundant database [36]. We selected only those that were uncontestably clear domain movements based on filtering criteria. We created two datasets, “Group 1” a strictly filtered group, and “Group 2” filtered based on more permissive criteria. Table 1 shows the filtering criteria for these two groups. We take the sequence of the Conformer 1 structure (the two structures submitted are assigned as “Conformer 1” and “Conformer 2” at the DynDom webserver by the expert user) with the residues annotated as hinge-bending or intradomain. Figure 1 shows glutamine binding protein, a typical member of Group 1. In the user-created set there is a great deal of redundancy. We follow Flores et al. [20] initially by filtering at 90% sequence identity on each group to ensure that no two sequences are selected for the same group if they have a sequence identity of 90% or higher. To achieve this we used the program CD-Hit [37]. The total counts for the data sets were 241 sequences in Group 1 and 372 sequences in Group 2. Group 1 can be regarded as containing clear hinge regions whereas Group 2 may contain some less hinge-like regions. Lists of the PDB structures in Groups 1 and 2 at 90% filtering are given in the Additional Data 1. These pairs identify the domain movement which can be viewed at the DynDom website.

We also filtered the datasets at 40 and 20% sequence identity thresholds using CD-Hit to assess the effect of removing homologous proteins. In the Results section we refer to the different datasets as Group1_90%, Group2_90%, Group1_40% and Group1_20%.

### Hinge Index

Flores et al. [20] proposed the Hinge Index, *HI*(*a*), for a given amino acid, *a*, as:

1 $$ HI(a)=\log \left(\frac{p\left(a|h\right)}{p(a)}\right), $$

which, is the log-likelihood ratio for the occurrence of amino acid *a* in a hinge region to its occurrence in the population as a whole. It is a measure of the propensity of an amino acid for a hinge region. *p*(*a*) is the probability of amino acid *a* irrespective of region and *p*(*a*| *h*) is the probability of amino acid *a* given it is in a hinge region, *h*. These probabilities were estimated from frequencies calculated using the annotated sequence data. Significance testing of *HI*(*a*) is performed using the hypergeometric distribution as outlined in detail by Flores et al. pages 6–7. The null hypothesis is that the observed number of occurrences of an amino acid of a particular type in hinge regions is the result of the random assignment of that amino acid to hinge regions according to its probability of occurrence in any region derived from its overall frequency. The alternative hypothesis is that it is not a random assignment with probabilities derived from their overall frequencies. Following Flores et al., the null hypothesis is rejected when *p* < 0.05.

### Kernel logistic regression

To build the training and test data sets from the sequence and bending region data, a sliding window of length *w* residues was placed over each sequence, resulting in subsequences of length *w* residues. If *w* is odd then the central residue of the window can either be in an intradomain region or a hinge-bending region. To get from our windowed sequence to a suitable input vector we employ “one-of-n-encoding”. For each window *i* the sequence is encoded as a 24*w* component input vector, ***x***_*i*_*,* where for each position in the window, 24 rows are assigned, each of which corresponds to the one of 24 “characters” in our alphabet: one character for each of the 20 standard amino acids plus “B”, “X” and “Z”, standing for ambiguous amino acids and “-” as a dummy character for those positions in the window that are beyond a terminus. The value of each of the 24 rows is set to 0 for each residue apart from the row of the residue at the corresponding window position which is set to 1.

Those windows with the central residue in an intradomain region were negatively labelled and have a target value for KLR of *t*_*i*_ = 0, and those with the central residue in a hinge-bending region were positively labelled and given a target value of *t*_*i*_ = 1. The number of negatively labelled records in the training set greatly outnumbered the number of positively labelled records, so this ratio in the training set was altered by randomly discarding negatively labelled examples. We elected to use a 1:9 proportion for the positive to negative cases for all training sets. In the Results section we show that variation of the proportion of positive to negative cases in the training set did not affect the AUROC.

KLR was applied to the data using UEA’s MATLAB Generalized Kernel Machine toolbox [38]. KLR [39] constructs a model of the form:

2 $$ \mathrm{logit}\left\{y\left(\mathbf{x}\right)\right\}=\mathbf{w}\bullet \boldsymbol{\phi} \left(\mathbf{x}\right)+b,\mathrm{where}\ \mathrm{logit}\left\{p\right\}=\log \left\{\frac{p}{1-p}\right\}, $$

where *b* is a scalar bias parameter, **w** is a vector of primal model parameters, and ***ϕ***(**x**) is the representation of **x** in a fixed feature space. The logit link function constrains the output of the model to lie between zero and one. Viewing this output as an *a-posteriori* probability of belonging to the “hinge” class, we classify test residues as part of a hinge-bending region if the output is above a certain threshold, and part of an intradomain region if the output is below the threshold.

Rather than define the non-linear transformation, ***ϕ***(**x**), directly, it is implicitly defined by a kernel function, $\mathcal{K}$, giving the inner product between vectors in the feature space,

3 $$ \mathcal{K}\left(\mathbf{x},{\mathbf{x}}^{\prime}\right)=\boldsymbol{\phi} \left(\mathbf{x}\right)\bullet \boldsymbol{\phi} \left({\mathbf{x}}^{\prime}\right), $$

where **x** and **x**^′^ are arbitrary vectors in the input space. A valid kernel function is one that obeys Mercer’s conditions; i.e. the resulting kernel matrix, **K,** is positive semi-definite for any set of points in the input space. We used three kernels starting with the linear kernel function, a straightforward scalar product of the input vectors:

4 $$ \mathcal{K}\left(\mathbf{x},{\mathbf{x}}^{\prime}\right)=\mathbf{x}\bullet {\mathbf{x}}^{\prime }. $$

The polynomial kernel of Eq. 5, maps the input vector into a higher dimensional feature space where new features are created from all monomials of order *d* or less of the original features. This allows non-linear separations of the data without requiring an enumeration of the possible combinations.

5 $$ \mathcal{K}\left(\mathbf{x},{\mathbf{x}}^{\prime}\right)={\left(\mathbf{x}\bullet \mathbf{x}\prime +c\right)}^d. $$

In this study, the kernel parameter *d* was set at two (for a quadratic kernel) or three (for a cubic kernel), and *c* is a hyper-parameter. The final kernel function used was the radial basis function (RBF) kernel:

6 $$ \mathcal{K}\left(\mathbf{x},{\mathbf{x}}^{\prime}\right)=\exp \left\{-\theta {\left\Vert \mathbf{x}-{\mathbf{x}}^{\prime}\right\Vert}^2\right\}, $$

where *θ* is a hyper-parameter controlling the sensitivity of the kernel.

Assume we are given a training set of *ℓ* examples, where **x**_*i*_ represents an input vector and *t*_*i*_ and *y*_*i*_ are, respectively, the expected and predicted outcome for the *i*^th^ training example. The optimal values of the primal model parameters, **w**, and bias, *b*, are found using the iteratively reweighted least squares training procedure [40] to minimise a regularised “cross-entropy” cost function:

7 $$ E=\frac{1}{2}{\left\Vert \mathbf{w}\right\Vert}^2-\frac{\gamma }{2}{\sum}_{i=1}^{\ell}\left[{t}_i\log \left\{{y}_i\right\}+\left(1-{t}_i\right)\log \left\{1-{y}_i\right\}\right]. $$

This optimisation problem is more conveniently solved in the dual representation, where the primal parameters are expressed in terms of the dual parameters:

8 $$ \mathbf{w}={\sum}_{i=1}^{\ell }{\alpha}_i\boldsymbol{\phi} \left({\mathbf{x}}_i\right)\ \mathrm{and}\ {\left\Vert \mathbf{w}\right\Vert}^2={\boldsymbol{\alpha}}^{\mathrm{T}}\mathbf{K}\boldsymbol{\alpha }, $$

where ***α*** is vector of dual model parameters. From Eq. 2, Eq. 3 and Eq. 8, the equation used to calculate an expected outcome from an input vector is:

9 $$ \mathrm{logit}\left\{y\left(\mathbf{x}\right)\right\}={\sum}_{i=1}^{\ell }{\alpha}_i\mathcal{K}\left({\mathbf{x}}_i,\mathbf{x}\right)+b. $$

The regularization parameter, *γ*, in Eq. 7 along with other hyper-parameters such as the kernel parameter *θ* in Eq. 6 and the polynomial kernel’s hyper-parameter *c* in Eq. 5, are tuned using the Nelder-Mead simplex algorithm [41] to minimise an approximate leave-one-out cross-validation estimate of the cross-entropy loss [40], which can be computed efficiently as a by-product of the training procedure, i.e. the leave-one-out cross-validation is performed on the training set.

## Supplementary information

**Additional file 1.** Data formatted list of PDB accession codes and chain IDs of pairs of structures used in Groups 1 and 2.

**Additional file 2: Table S1.** Table giving matrix of *p*-values for the pairwise comparisons of the AUROC for the linear, quadratic, cubic and RBF models for Group1_90% dataset.

**Additional file 3: Table S2.** Table giving matrix of *p*-values for the pairwise comparisons of the AUROC for the linear, quadratic and cubic models for Group2_90% dataset.

**Additional file 4: Table S3.** Table for comparison of AUROCs for window lengths 81 and 87.

**Additional file 5: Figure S1.** HingeIndex values for amino acids evaluated from Group2_90% dataset.

**Additional file 6: Figure S2.** (A) ROC curves for the quadratic model with window length 81 on Group1_90% with various proportions of positive to negative training examples. (B) Plots of the AUROC against proportion of positive to negative training examples for different window lengths.

**Additional file 7: Figure S3.** Precision-Recall curve for Group1_90%.

**Additional file 8: Figure S4.** Area under Precision-Recall curves for different KLR models at different window lengths for Group1_90% dataset.

**Additional file 9: Figure S5.** *p*-values at different window lengths for the Group1_40% dataset determined by doing a paired t-test of the AUROC between the linear and quadratic KLR models.

## Data Availability

All the data used are available from the Protein Data Bank (PDB) – see Additional Data 1 for list of accession codes – at wwpdb.org and from the DynDom website at www.cmp.uea.ac.uk/dyndom.

## Abbreviations

PDB: Protein Data Bank
KLR: Kernel logistic regression
DBMM: Database of macromolecular motion
HI: Hinge Index
ROC: Receiver operator characteristic
AUROC: Area under ROC curve
AUPRC: Area under precision-recall curve
