# Supplementary material for: Investigation of sequence features of hinge-bending regions in proteins with domain movements using kernel logistic regression
Source: BMC Bioinformatics. 2020 Apr 9;21:137. doi: 10.1186/s12859-020-3464-3 (PMC7147021; doi:10.1186/s12859-020-3464-3)
Supplement: Supplementary file 1 — Additional file 1. Data formatted list of PDB accession codes and chain IDs of pairs of structures used in Groups 1 and 2. [file 12859_2020_3464_MOESM1_ESM.pdf]

Additional Data 1

GROUP1 and GROUP2 datasets

Group 1

Protein Name	Conformer 1		Conformer 2	
	PDB Code	Chain ID	PDB Code	Chain ID
Dna-Directed RNA Polymerase II Largest Subunit	1i50	A	2nvq	A
Damage-Specific DNA Binding Protein 1	2b5m	A	2hye	A
Pullulanase	2yoc	A	2yoc	B
Glucansucrase	3klk	A	4amc	A
Ubiquitin-Activating Enzyme E1 1	4ii3	A	4ii2	A
Leucyl-Trna Synthetase	1wz2	B	1wkb	A
DNA Polymerase	1ig9	A	2dy4	C
Ns5 Polymerase	4k6m	A	5ccv	A
Pyruvate,orthophosphate Dikinase	1vbh	A	2r82	A
T7 Lysozyme	1h38	A	1s77	D
Importin Beta-1 Subunit	2bku	B	2bpt	A
Protein Translocase Subunit Seca	3jv2	A	1m6n	A
Isocitrate Dehydrogenase [Nadp]	3mbc	A	1j1w	A
Programmed Cell Death 6-Interacting Protein	2oev	A	4jjy	A
Lactoferrin	1bka	A	1cb6	A
Argonaute	3hk2	A	3f73	A
Atp-Dependent DNA Helicase Rep	1uaa	A	1uaa	B
Polyphosphate Kinase	2o8r	A	2o8r	B
Calpain 2, Large [Catalytic] Subunit Precursor	1u5i	A	2ary	A
Secreted Effector Protein	2qyu	A	2qza	A
Glucosamine-Fructose-6-Phosphate Aminotransferase	2j6h	A	1jxa	C
Protein Phosphatase Pp2a	2nym	D	2ie4	A
Flavocytochrome C Fumarate Reductase	1d4e	A	1qo8	A
Phosphoenolpyruvate-Protein Phosphotransferase	2hwg	A	1zym	A
Acyl-Coenzyme A Synthetase Acsm2a, Mitochondrial Precursor	3b7w	A	3c5e	A
Long Chain Fatty Acid-Coa Ligase	1ult	A	1ult	B
Chitin Oligosaccharide Binding Protein	1zu0	A	1zty	A
D-3-Phosphoglycerate Dehydrogenase	1ygy	B	1ygy	A
5''-Nucleotidase	1hp1	A	1hpu	C
Groel	1aon	H	2c7e	A
5''-Nucleotidase	4h2g	A	4h2i	A
Diphtheria Toxin	1f0l	B	1tox	B
Luciferase	1lci	A	2d1r	A
Pyruvate Kinase Isozymes M1/m2	4fxj	A	3srh	A

Periplasmic Oligopeptide-Binding Protein	1rkm	A	2rkm	A
Lethal Factor	1yqy	A	1jky	A
Nitrite Reductase	1hzv	A	1nir	B
Metabotropic Glutamate Receptor Subtype 1	2e4u	A	3sm9	A
2,3-Bisphosphoglycerate-Independent Phosphoglycerate Mutase	1o98	A	2ify	A
Ba3-Type Cytochrome-C Oxidase	2ify	A	4my4	A
Dipeptide-Binding Protein	1dpe	A	1dpp	A
4-Chlorobenzoyl Coa Ligase	3cw8	X	3cw9	A
Pyruvate Kinase	1pkl	A	3hqp	A
Nickel-Binding Periplasmic Protein	2noo	A	1zlq	B
Macromolecule-Binding Periplasmic Protein	1kwh	A	1j1n	B
Algq1	1y3q	A	1y3n	A
Fimbrin-Like Protein	1pxy	B	1pxy	A
Chaperone Protein Htpg	1y4s	B	2iop	A
Malonyl Coa Synthetase	4fut	A	4fuq	A
Intermedilysin	1s3r	A	4bik	A
2-Succinylbenzoate--Coa Ligase	5buq	B	5bur	A
Atp Synthase Beta Chain, Mitochondrial	2hld	N	2hld	M
F1-Atpase	1sky	E	1bmf	F
Son Of Sevenless Protein Homolog 1	1xd2	C	2ii0	A
Atp Synthase Subunit Beta	2jdi	E	1h8e	D
Metabotropic Glutamate Receptor Subtype 1	1ewk	B	1ewk	A
Udp-N-Acetylmuramate-L-Alanine Ligase	1p3d	A	1gqq	B
3-Phosphoshikimate 1-Carboxyvinyltransferase	2gg4	A	2gg6	A
Integrin Beta-3	1tye	B	1jv2	B
3-Phosphoshikimate 1-Carboxyvinyltransferase	3roi	A	3slh	A
Protein (Udp-N-Acetylmuramoyl-L-Alanine:d-Glutamate Ligase)	3uag	A	1e0d	A
5-Enolpyruvylshikimate-3-Phosphate Synthase	1rf6	C	1rf5	B
Type I Restriction-Modification Enzyme, S Subunit	1yf2	B	1yf2	A
Elongation Factor 1-Alpha	1jny	A	1f60	A
Isocitrate Dehydrogenase	1sjs	A	1hj6	A
3-Phosphoglycerate Kinase	13pk	A	1php	A
Isocitrate Dehydrogenase [Nadp] Cytoplasmic	1t09	A	1t0l	D
Isocitrate Dehydrogenase	1lwd	A	3mas	A
Putative Fimbrial Subunit	4hss	A	4hss	B
Gamma-Aminobutyric Acid Type B Receptor Subunit 1	4mqe	A	4ms3	A
Heat Shock Locus U	1do0	A	1do0	B
D-3-Phosphoglycerate Dehydrogenase (Phosphoglycerate 3 Dehydrogenase) (E.C.1.1.1.95)	1psd	A	1sc6	B
Molybdopterin Biosynthesis Protein Moea	2nqq	C	2nqq	A
47 Kda Membrane Antigen	1o75	B	1o75	A

Elongation Factor Tu	1ob5	C	2c78	A
Protein (Eukaryotic Peptide Chain Release Factor Subunit 1)	1dt9	A	3e1y	A
Aminopeptidase T	2ayi	D	2ayi	B
Succinyl-Coa Synthetase, Beta Chain	1eud	B	2fp4	B
Acarbose/maltose Binding Protein Gach	3k01	A	3jzj	A
Polymerase (Dna Directed) Kappa	1t94	B	2oh2	B
Molybdopterin Biosynthesis Moea Protein	1wu2	A	1wu2	B
Udp-N-Acetylglucosamine 2-Epimerase	3beo	A	1o6c	A
Actin	2zwh	A	1j6z	A
Maltose Abc Transporter, Periplasmic Maltose-Binding Protein	2gha	A	2ghb	B
D-Maltodextrin Binding Protein	1anf	A	1jw5	A
Chemotaxis Protein Chea	1b3q	A	2ch4	A
Collybistin II	2dfk	A	2dfk	C
Rho Guanine Nucleotide Exchange Factor 12	1x86	A	1txd	A
Uncharacterized Protein	4xe8	A	4xe7	A
Udp-N-Acetylglucosamine 2-Epimerase	4neq	A	4nes	A
3-Isopropylmalate Dehydrogenase	2y3z	A	4f7i	A
Choline Kinase Alpha	2ckq	B	2i7q	A
Dbh Protein	1k1q	A	2rdi	A
Leu/ile/val-Binding Protein	1z15	A	1z16	A
Nagk Protein	2ch6	D	2ch6	B
DNA Polymerase IV	3qz7	A	3bq1	A
Purine Nucleotide Synthesis Repressor	1jft	A	1dbq	A
Atp-Dependent Hsl Protease Atp-Binding Subunit Hslu	1im2	A	1qg4	A
Twitching Motility Protein Pilt	2gsz	A	2gsz	E
DNA Polymerase III, Delta Subunit	1jqj	D	1xxh	F
D-Lactate Dehydrogenase	1j49	B	1j4a	D
Ovotransferrin	1tfa	A	1iej	A
Rfcs	1iqp	C	1iqp	D
DNA Polymerase Beta	1bpd	A	2bpg	B
Igg Heavy Chain	1za6	B	1za6	D
Serotransferrin	1ryo	A	1bp5	C
Atp-Dependent Clp Protease Atp-Binding Subunit Clpx	3hws	A	3hws	B
Parm	1mwk	A	1mwm	A
M-Calpain	1kxr	A	1ziv	A
Thioredoxin Reductase	1tde	A	1f6m	A
Translation Initiation Factor Eif-2b, Delta Subunit	3a9c	A	3vm6	A
Xylanase J	2dck	A	2dcj	A
Calpain 9	1ziv	A	2p0r	A
Ig Epsilon Chain C Region	4j4p	B	1o0v	B
Spectrin Alpha Chain, Brain	1u4q	B	1cun	B

Sugar Transport Protein	1tjy	A	1tm2	A
Nuclear Factor Nf-Kappa-B P105 Subunit	1ooa	A	2i9t	B
Manganese-Dependent Inorganic Pyrophosphatase	1k20	B	1k23	B
Interleukin-1 Receptor	1itb	B	1g0y	R
Manganese-Dependent Inorganic Pyrophosphatase	1k23	A	1wpm	A
Guanine Nucleotide Exchange Factor Dbs [Fragment]	1rj2	G	1rj2	J
D-Galactose-Binding Periplasmic Protein	2fw0	A	2hph	A
Sugar Abc Transporter, Periplasmic Sugar-Binding Protein	3c6q	A	3c6q	C
D-3-Phosphoglycerate Dehydrogenase, Putative	4nfy	A	4njm	A
Nf-Kappa-B P65	1nfi	C	2ram	B
Mrna Decapping Enzyme	1xmm	B	1xml	B
Probable Transcriptional Regulator	2esn	A	2esn	C
Virb11 Homolog	1nlz	F	1nlz	E
Hexokinase	2e2n	A	2e2o	A
Type IIe Restriction Endonuclease Naei	1ev7	A	1iaw	A
2-Dehydropantoate 2-Reductase	1ks9	A	2ofp	B
Ribose Abc Transporter, Periplasmic Ribose-Binding Protein	2fn9	A	2fn8	A
Titin	2ill	A	2nzi	B
Serine/threonine-Protein Kinase Pak 4	2cdz	A	2c30	A
D-Allose-Binding Periplasmic Protein	1gub	A	1rpj	A
Glutamate [Nmda] Receptor Subunit Zeta 1	1y20	A	1pbq	A
Nuclear Factor Of Activated T-Cells, Cytoplasmic 2	1owr	Q	1owr	M
Potassium Channel	2wln	A	2wlk	A
Pantothenate Synthetase	3ag5	A	3ag6	A
Diaminopimelate Epimerase	2q9h	A	2gke	A
Mhc Class I H-2dd Heavy Chain	1qo3	A	1ddh	A
Glutamate Receptor Ionotropic, Nmda 2A	4nf5	B	3oel	A
D-Ribose-Binding Protein	1ba2	A	1urp	C
Osmoprotection Protein (Prox)	1sw4	A	1sw5	C
Udp-2,3-Diacylglucosamine Pyrophosphatase Lpxi	4ggm	X	4j6e	A
N-Methyl-D-Aspartate Receptor Subunit 1	1pbq	B	1pb7	A
Pectocin M2	4n58	A	4n59	A
Spac19a8.12 Protein	2qkm	B	2qkm	D
Phosphate-Binding Protein Psts 1	4exl	A	4lat	A
Probable Translation Initiation Factor 2 Alpha Subunit	1yz6	A	1yz7	A
Glutamate Receptor Subunit 2	1ftj	B	1fto	A
Glutamate Receptor 3	3dln	A	1fto	A
Acetylglutamate Kinase	2wxb	A	1gs5	A

Glutamate Receptor, Ionotropic Kainate 1	2f34	B	1ycj	B
Betaine Abc Transporter Permease And Substrate Binding Protein	3l6g	A	3l6h	A
Glutamate Receptor, Ionotropic Kainate 1	1s7y	B	1fto	A
Vinculin Isoform Vcl	1ydi	A	1rke	A
Glutamate Receptor, Ionotropic Kainate 3	4e0w	A	1fto	A
Nopaline-Binding Periplasmic Protein	4pow	A	4p0i	A
Angiostatin	1ki0	A	2doh	X
Endonuclease VIII	1k3x	A	1q3b	A
Major Surface Antigen P30	1kzq	A	1ynt	G
Alpha-1 Catenin	1h6g	A	1l7c	C
Mg2+ Transporter Mgte	2yvy	A	2yvz	A
Tight Junction Protein Zo-1	3lh5	A	3kfv	A
Replication Protein A 70 Kda Dna-Binding Subunit	1fgu	B	1fgu	A
Dihydrodipicolinate Reductase	1yl7	C	1p9l	A
Hypothetical Protein	2i76	A	2i76	B
Dihydrodipicolinate Reductase	3qy9	B	3qy9	D
Putative Abc Transporter, Periplasmic Binding Protein, Amino Acid	2yln	A	3zsf	A
Lysine, Arginine, Ornithine-Binding Protein	2lao	A	1lst	A
Glutamate Receptor Delta-2 Subunit	2v3t	A	2v3u	A
Windbeutel Protein	2c1y	A	2c0e	A
Dna-Directed RNA Polymerase Alpha Chain	1ynj	A	1ynn	B
Dna-Directed RNA Polymerase Alpha Chain	2a6h	A	1iw7	L
Fab 17B Heavy Chain	1rz8	B	2i60	R
Igg1 Antibody 58.2 (Heavy Chain)	1f58	H	3f58	H
Abc-Type Transporter, Periplasmic Subunit Family 3	4psh	A	4prs	A
Fab-Ysd1 Heavy Chain	1za3	H	1za3	B
Glutamine Binding Protein	1wdn	A	1ggg	B
Hyb3 Heavy Chain	1w72	H	1dfb	H
Calcium-Gated Potassium Channel Mthk	2fy8	C	2fy8	H
Chimera Of Fab2c4: "Humanized" Murine Monoclonal Antibody	1l7i	H	1s78	F
Igg1 Fab Fragment	1igc	H	2aab	H
Catalytic Elimination Antibody 13G5 Heavy Chain	2gjz	B	2gk0	H
Chimeric Germline Precursor Of Oxy-Cope Catalytic Antibody Az-28 (Heavy Chain)	1d5i	H	1d5b	B
Fab Fragment, Heavy Chain	2h2s	E	2htl	C
Igg1 Fab Fragment (Hc19)	1gig	H	2vir	B
Antibody Light Chain	1jgu	L	1baf	L
Fab Fragment, Antibody A5b7	1ad0	B	1rmf	H
Igg Heavy Chain	2dd8	H	1rzi	F
Fab Fragment Of 8F5 Antibody Against Human Rhinovirus 3 Serotype 2 4	1bbd	L	1hin	L

K42-41l Fab Light Chain	1mju	L	1uz8	A
28B4 Fab	1kem	H	1q9l	B
Pc283 Immunoglobulin	1kcr	H	1kcu	H
Anti-Idiotypic Monoclonal Antibody (Light Chain)	2aab	L	1iqw	L
Igg 5C8	15c8	H	1fgn	H
Antibody M41	1gpo	L	1keg	L
Fab Heavy Chain	1xf3	H	1i8m	B
Immunoglobulin Gamma-1 Heavy Chain Constant Region	1e4k	B	2iwg	A
Igg1 Antibody 58.2 (Light Chain)	1f58	L	3f58	L
Immunoglobulin G1 (Igg1)	2mcg	1	2mcg	2
Loc - Lambda 1 Type Light-Chain Dimer	3bjl	B	1bjm	A
Mature Metal Chelatase Catalytic Antibody, Heavy Chain	1ngy	B	1n7m	L
Dihydrodipicolinate Reductase	1vm6	C	1vm6	B
Igg2a Fab Fragment (50.1)	1ggi	M	1ai1	L
Igm-Kappa Cold Agglutinin (Light Chain)	1dn0	C	1rhh	A
Immunoglobulin Lambda Light Chain	1jvk	B	1jvk	A
Monoclonal Antibody 2D12.5, Igg1 Gamma Heavy Chain	1gig	L	1q0x	L
Antibody Light Chain 11K2	2bdn	L	1osp	L
Immunoglobulin 48G7 Germline Fab	1gaf	L	1gpo	L
Fab 17B Light Chain	1rz8	A	2ny1	C
Fab Fragment Of Murine Monoclonal Antibody An02 Complex 3 With Its Hapten (2,2,6,6-Tetramethyl-1-Piperidinyloxy- 4 Dinitrophenyl)	1baf	L	1cz8	L
33H1 Fab Light Chain	1ors	A	1fig	L
Humanized Antibody Hfe7a, Light Chain	1it9	L	2gcy	A
Igg Antibody (Light Chain)	1emt	L	2a6i	A
Igg2b (Kappa)	1cgs	H	2cgr	H
Monoclonal Anti-Estradiol 10G6d6 Immunoglobulin Gamma-1 Chain	1jn6	B	1jnh	B
Pc287 Immunoglobulin	1kcu	L	1fsk	K
Antibody Light Chain Fab	1i8m	A	1qbm	L
Erythropoietin Receptor	1eer	B	1ern	B
17E8	1eap	A	1a0q	L
Fab E51 Light Chain	1rzf	L	1q1j	M
Fab Fragment, Antibody A5b7	1ad0	A	1rmf	L
Germline Metal Chelatase Catalytic Antibody, Chain H	1n7m	H	1ngy	A
Humanized Antibody D3h44	1pg7	H	1jps	H
Immunoglobulin	1ce1	L	1t04	C
Lambda III Bence Jones Protein Cle	1lil	A	1lil	B
Chimeric Germline Precursor Of Oxy-Cope Catalytic Antibody Az-28 (Light Chain)	1d6v	L	1axs	L
Hyb3 Light Chain	1w72	M	1adq	L

Fibroblast Growth Factor Receptor 2	1e0o	D	1djs	A
Septum Site-Determining Protein Minc	1hf2	C	1hf2	A
Vascular Cell Adhesion Molecule-1	1vsc	B	1vca	A
P58-Cl42 Kir	1nkr	A	2dli	A
N2b-Titin Isoform	2f8v	C	2a38	C
Transcriptional Regulator, Tetr Family	1zkg	A	1z77	A
Hypothetical Transcriptional Regulator In Qaca 5''Region	1jt0	A	1jtx	A
Muscle-Specific Kinase Receptor	2iep	B	2iep	A
Transcriptional Regulator	3vok	A	3vp5	A
Tenascin	1qr4	B	1qr4	A
Fatty Acid-Binding Protein, Epidermal	4azr	B	1b56	A

Group 2

	Conformer 1		Conformer 2	
Protein Name	PDB Code	Chain ID	PDB Code	Chain ID
Dna-Directed RNA Polymerase II Largest Subunit	1i50	A	1y1w	A
Damage-Specific DNA Binding Protein 1	2b5m	A	2hye	A
Pullulanase	2yoc	A	2yoc	B
Glucansucrase	3klk	A	4amc	A
Sarcoplasmic/endoplasmic Reticulum Calcium Atpase 1	2c9m	A	2c9m	B
Vinculin Isoform 1	1tr2	A	1st6	A
Ubiquitin-Activating Enzyme E1 1	4ii3	A	4ii2	A
Leucyl-Trna Synthetase	1wz2	B	1wkb	A
DNA Polymerase	1waf	B	1ig9	A
Ns5 Polymerase	4k6m	A	5ccv	A
T7 Lysozyme	1h38	A	1s77	D
Pyruvate,orthophosphate Dikinase	1vbh	A	2r82	A
Importin Beta-1 Subunit	2bku	B	2bpt	A
Clpb Protein	1qvr	C	1qvr	A
Myosin Heavy Chain	1dfl	A	1sr6	A
Preprotein Translocase Seca Subunit	1tf5	A	3jv2	A
Putative Secreted Lyase	2wda	A	2x03	B
Isocitrate Dehydrogenase [Nadp]	3mbc	A	1j1w	A
Dipeptidyl-Peptidase 3	3fvy	A	3t6b	A
Programmed Cell Death 6-Interacting Protein	2oev	A	4jjy	A
Argonaute	2nub	A	2f8s	A
Lactoferrin	1bka	A	1cb6	A
Argonaute	3hk2	A	3f73	A
Protease/helicase Ns3	1a1v	A	8ohm	A
Atp-Dependent DNA Helicase Rep	1uaa	A	1uaa	B
Polyphosphate Kinase	2o8r	A	2o8r	B
Calpain 2, Large [Catalytic] Subunit Precursor	1u5i	A	2ary	A
Secreted Effector Protein	2qyu	A	2qza	A
Receptor Tyrosine-Protein Kinase Erbb-4	2ahx	B	1yy9	A

Regulator Of Nonsense Transcripts 1	2gjk	A	2gk6	B
Epidermal Growth Factor Receptor	1yy9	A	1m6b	A
Glucosamine-Fructose-6-Phosphate Aminotransferase	2j6h	A	1jxa	C
Dengue 4 Ns3 Full-Length Protein	2vbc	A	2whx	A
Penicillin-Binding Protein 2	2olu	A	2olv	B
Protooncoprotein	1n8y	C	1n8z	C
Acylamino-Acid-Releasing Enzyme	3o4j	A	3o4j	B
Atp Sulfurylase	1i2d	C	1m8p	A
Phosphoglucomutase 1	1kfi	A	1kfq	B
Protein Phosphatase Pp2a	2nym	D	2ie4	A
Chitinase A	3b8s	B	3b9d	A
Flavocytochrome C Fumarate Reductase	1d4e	A	1qo8	A
Hiv-1 Reverse Transcriptase	1hvu	G	1vrt	A
Phosphoenolpyruvate-Protein Phosphotransferase	2hwg	A	1zym	A
Sumo-Activating Enzyme Subunit 2	3kyc	B	3kyd	B
Acyl-Coenzyme A Synthetase Acsm2a, Mitochondrial Precursor	3b7w	A	3c5e	A
Long Chain Fatty Acid-Coa Ligase	1ult	A	1ult	B
Chitin Oligosaccharide Binding Protein	1zu0	A	1zty	A
D-3-Phosphoglycerate Dehydrogenase	1ygy	B	1ygy	A
5''-Nucleotidase	1hp1	A	1hpu	C
Groel	1aon	H	2c7e	A
5''-Nucleotidase	4h2f	A	4h2i	A
Diphtheria Toxin	1f0l	B	1tox	B
Luciferase	1lci	A	2d1r	A
Pyruvate Kinase	2g50	E	1aqf	H
Thermosome Alpha Subunit	1q3q	A	3ko1	A
Periplasmic Oligopeptide-Binding Protein	1rkm	A	2rkm	A
Phosphoenolpyruvate Carboxykinase [Atp]	1ytm	B	1yvy	A
Exodeoxyribonuclease V, Subunit Recd, Putative	3gpl	A	3gp8	A
Lethal Factor	1yqy	A	1jky	A
Nitrite Reductase	1hzv	A	1nir	B
Metabotropic Glutamate Receptor Subtype 1	2e4u	A	3sm9	A
2,3-Bisphosphoglycerate-Independent Phosphoglycerate Mutase	1o98	A	2ify	A
Ba3-Type Cytochrome-C Oxidase	2ify	A	4my4	A
Dipeptide-Binding Protein	1dpe	A	1dpp	A
4-Chlorobenzoyl Coa Ligase	3cw8	X	3cw9	A
Exocyst Complex Component Exo70	2b7m	A	2b1e	A
Protein (Pyruvate Kinase)	1pkl	B	1pkl	H
Nickel-Binding Periplasmic Protein	2noo	A	1zlq	B
Macromolecule-Binding Periplasmic Protein	1kwh	A	1j1n	B
Algq1	1y3q	A	1y3n	A
Benzoate-Coenzyme A Ligase	2v7b	A	4eat	A
Fimbrin-Like Protein	1pxy	B	1pxy	A
Chaperone Protein Htpg	1y4s	B	2iop	A
Glycogen Synthase	3d1j	A	2qzs	A

Malonyl Coa Synthetase	4fut	A	4fuq	A
Intermedilysin	1s3r	A	1s3r	B
2-Succinylbenzoate--Coa Ligase	5buq	B	5bur	A
Atp Synthase Beta Chain, Mitochondrial	2hld	N	2hld	M
F1-Atpase	1sky	E	1bmf	F
Son Of Sevenless Protein Homolog 1	1xd2	C	2ii0	A
S-Adenosyl-L-Homocysteine Hydrolase (Sahase)	4lvc	A	4lvc	D
Atp Synthase Subunit Beta	2jdi	E	1h8e	D
Metabotropic Glutamate Receptor Subtype 1	1ewk	B	1ewk	A
Tyrosine Phenol-Lyase	2ez2	A	2ez2	B
Glutamate Dehydrogenase	1hrd	A	1bgv	A
Udp-N-Acetylmuramate-L-Alanine Ligase	1p3d	A	1gqq	B
3-Phosphoshikimate 1-Carboxyvinyltransferase	2gg4	A	2gg6	A
Integrin Beta-3	1tye	B	1jv2	B
Pyrimidine Nucleoside Phosphorylase	1brw	B	1brw	A
3-Phosphoshikimate 1-Carboxyvinyltransferase	3roi	A	3slh	A
Protein (Udp-N-Acetylmuramoyl-L-Alanine:d-Glutamate Ligase)	3uag	A	1e0d	A
S-Adenosylhomocysteine Hydrolase	1b3r	A	1k0u	A
5-Enolpyruvylshikimate-3-Phosphate Synthase	1rf6	C	1rf5	B
Type I Restriction-Modification Enzyme, S Subunit	1yf2	B	1yf2	A
Isocitrate Dehydrogenase	1xkd	B	1xkd	A
Udp-N-Acetylglucosamine Enolpyruvyl Transferase	1ejd	B	1q3g	A
Elongation Factor 1-Alpha	1jny	A	1f60	A
Isocitrate Dehydrogenase	1sjs	A	4icd	A
3-Phosphoglycerate Kinase	13pk	A	1php	A
Isocitrate Dehydrogenase [Nadp] Cytoplasmic	1t09	A	1t0l	D
Isocitrate Dehydrogenase	1lwd	A	3mas	A
Phosphoglycerate Kinase, Testis Specific	2paa	A	2x15	A
Putative Fimbrial Subunit	4hss	A	4hss	B
Folylpolyglutamate Synthase	1jbw	A	2gc5	A
Gamma-Aminobutyric Acid Type B Receptor Subunit 1	4mqe	A	4ms3	A
Atrial Natriuretic Peptide Clearance Receptor	1jdn	A	1jdp	A
Heat Shock Locus U	1do0	A	1do0	B
D-3-Phosphoglycerate Dehydrogenase (Phosphoglycerate 3 Dehydrogenase) (E.C.1.1.1.95)	1psd	A	1sc6	B
Elongation Factor Tu	1tui	A	1ha3	A
Molybdopterin Biosynthesis Protein Moea	2nqq	C	2nqq	A
Phosphoglycerate Kinase 1	3c3b	A	2zgv	A
Polyprotein	2i69	A	2hg0	A
47 Kda Membrane Antigen	1o75	B	1o75	A
Acetate Kinase	1tuu	A	1g99	B
Protein (Eukaryotic Peptide Chain Release Factor Subunit 1)	1dt9	A	3e1y	A
Aminopeptidase T	2ayi	D	2ayi	B
Aspartate Aminotransferase	1arg	A	9aat	A

Binding Protein Component Of Abc Sugar Transporter	5dvi	A	5dvf	A
Abc Transporter Binding Protein Acbh	3ooa	A	3oo6	A
Mandelate Racemase/muconate Lactonizing Enzyme/enolase Superfamily	2hzg	A	2hzg	B
Succinyl-Coa Synthetase, Beta Chain	1eud	B	2fp4	B
Acarbose/maltose Binding Protein Gach	3k01	A	3jzj	A
DNA Gyrase B	1ei1	A	1kij	A
Glycosyltransferase Gtfa	1pn3	A	1pn3	B
A2,3-Sialyltransferase, A2,6-Sialyltransferase	2ex0	B	2ihz	A
Sugar Abc Transporter, Sugar-Binding Protein	2hq0	A	2i58	A
Structural Polyprotein (P130)	2ala	A	3n41	F
Mrna Capping Enzyme Alpha Subunit	1p16	A	1p16	B
Polymerase (Dna Directed) Kappa	1t94	B	2oh2	B
Protein (Arrestin)	1cf1	C	4j2q	A
Pentafunctional Arom Polypeptide	1sg6	B	1dqs	B
D-3-Phosphoglycerate Dehydrogenase (Phosphoglycerate 1Psd 3 Dehydrogenase) (E.C.1.1.1.95)	1sc6	B	1sc6	D
Molybdopterin Biosynthesis Moea Protein	1wu2	A	1wu2	B
Udp-N-Acetylglucosamine 2-Epimerase	3beo	A	1o6c	A
Beta-Actin	1nwk	A	2zwh	A
Maltose Abc Transporter, Periplasmic Maltose-Binding Protein	2gha	A	2ghb	B
Mlc Protein	1z6r	A	1z6r	B
Probable Butyrate Kinase 2	1saz	A	1x9j	D
D-Maltodextrin Binding Protein	1omp	A	3mbp	A
Alanine Dehydrogenase	2vhx	A	2vhx	F
Chemotaxis Protein Chea	1b3q	A	2ch4	A
Atp-Dependent RNA Helicase Eif4a	2vso	A	2j0u	A
Collybistin II	2dfk	A	2dfk	C
Udp-N-Acetylglucosamine 2-Epimerase	1vgv	D	1vgv	B
Udp-N-Acetylglucosamine 2-Epimerase	1v4v	A	1v4v	B
Rho Guanine Nucleotide Exchange Factor 12	1x86	A	1txd	A
Uncharacterized Protein	4xe8	A	4xe7	A
B-Cell Mitogen	1w62	B	1w61	B
Udp-N-Acetylglucosamine 2-Epimerase	4neq	A	4nes	A
3-Isopropylmalate Dehydrogenase	1ipd	A	1a05	A
Choline Kinase Alpha	2ckq	B	2i7q	A
Putative Abc-Transporter Atp-Binding Protein	1z47	A	1z47	B
Autoinducer 2-Binding Periplasmic Protein Luxp	1zhh	A	1jx6	A
Dbh Protein	1k1q	A	3bq1	A
Leu/ile/val-Binding Protein	1z15	A	1z16	A
Protein Kinase Inhibitor	1cdk	A	1ctp	E
Nagk Protein	2ch6	D	2ch6	B
Glycine Receptor Subunit Alphaz1	3jad	A	3jae	A
DNA Polymerase IV	3qz7	A	3bq1	A

Avermectin-Sensitive Glutamate-Gated Chloride Channel Glucl Alpha	3rhw	A	4tnv	A
Purine Nucleotide Synthesis Repressor	1jft	A	1dbq	A
Trna Pseudouridine Synthase D	1si7	A	1szw	A
DNA Polymerase III, Delta Subunit	1jqj	D	1xxh	F
Atp-Dependent Hsl Protease Atp-Binding Subunit Hslu	1im2	A	1qg4	A
Tight Junction Protein Zo-1	3lh5	A	3kfv	A
Twitching Motility Protein Pilt	2eww	A	2gsz	A
D-Lactate Dehydrogenase	1j49	B	1j4a	D
Phosphate Acetyltransferase	1qzt	B	2af3	C
Protein Rdmb	1xds	A	1qzz	A
Thioredoxin Reductase	2zbw	A	2zbw	B
Anthranilate Phosphoribosyltransferase	2elc	A	2elc	C
Ovotransferrin	1tfa	A	1iej	A
Serotransferrin	1ryo	A	1bp5	C
Plasmepsin	1qs8	B	1miq	B
Rfcs	1iqp	C	1iqp	D
Tryptophanyl-Trna Synthetase	1maw	A	1mau	A
DNA Polymerase Beta	1bpd	A	2bpg	B
Igg Heavy Chain	1za6	B	1za6	D
Recombinase Cre	1pvp	A	1q3u	F
4-Hydroxythreonine-4-Phosphate Dehydrogenase	1ptm	B	1r8k	A
Phosphate-Binding Protein	1oib	A	1a55	A
Atp-Dependent Clp Protease Atp-Binding Subunit Clpx	3hws	A	3hws	B
Diaminopimelic Acid Dehydrogenase	3dap	A	1dap	B
Parm	1mwk	A	1mwm	A
M-Calpain	1kxr	A	1ziv	A
Thioredoxin Reductase	1tde	A	1f6m	A
Translation Initiation Factor Eif-2b, Delta Subunit	3a9c	A	3vm6	A
Xylanase J	2dck	A	2dcj	A
Calpain 9	1ziv	A	2p0r	A
Ige Heavy Chain Epsilon-1	1fp5	A	1f6a	B
Guanine Nucleotide Exchange Factor Dbs [Fragment]	1rj2	G	1rj2	J
Iron Binding Protein Fbpa	1si1	A	1q35	A
Spectrin Alpha Chain, Brain	1u4q	B	1cun	B
Virb11 Homolog	1nlz	F	1nlz	E
Sugar Transport Protein	1tjy	A	1tm2	A
Glucokinase	1q18	A	1sz2	A
Nuclear Factor Nf-Kappa-B P105 Subunit	1ooa	A	2i9t	B
Interleukin-1 Receptor, Type I	1g0y	R	1ira	Y
Manganese-Dependent Inorganic Pyrophosphatase	1k20	B	1wpp	A
Probable Hpr(ser) Kinase/phosphatase	1knx	B	1knx	E
Ferric-Iron Binding Protein	1r1n	A	1d9y	A
Iron-Utilization Periplasmic Protein	1d9v	A	1mrp	A
Manganese-Dependent Inorganic Pyrophosphatase	1k23	A	1wpm	A

Cell Division Protein Ftsz	3voa	A	1w5a	A
Protein Hi0146	2cey	A	2cex	B
D-Galactose-Binding Periplasmic Protein	2fw0	A	2hph	A
Sugar Abc Transporter, Periplasmic Sugar-Binding Protein	3c6q	A	3c6q	C
Phosphoglycerate Dehydrogenase	1wwk	A	2ekl	A
Probable Manganese-Dependent Inorganic Pyrophosphatase	1i74	A	1i74	B
D-3-Phosphoglycerate Dehydrogenase, Putative	4nfy	A	4njm	A
Mycothiol Synthase	2c27	A	1p0h	A
Nf-Kappa-B P65	1nfi	C	2ram	B
Mrna Decapping Enzyme	1xmm	B	1xml	B
Probable Transcriptional Regulator	2esn	A	2esn	C
Hexokinase	2e2n	A	2e2o	A
Peroxisomal Targeting Signal 1 Receptor	2c0m	A	2c0l	A
Type IIe Restriction Endonuclease Naei	1ev7	A	1iaw	A
Aspartate Carbamoyltransferase	1gq3	A	1gq3	C
2-Dehydropantoate 2-Reductase	1ks9	A	2ofp	B
Lysr-Type Regulatory Protein	1iz1	A	1iz1	B
Ribose Abc Transporter, Periplasmic Ribose- Binding Protein	2fn9	A	2fn8	A
Titin	2ill	A	2nzi	B
L-3-Hydroxyacyl-Coa Dehydrogenase	1f14	B	1f0y	B
Serine/threonine-Protein Kinase Pak 4	2cdz	A	2c30	A
D-Allose-Binding Periplasmic Protein	1gub	A	1rpj	A
Haloacid Dehalogenase-Like Hydrolase	4qjb	A	4qjb	B
Epidermal Growth Factor Receptor	4i1z	A	4r3r	A
Glutamate [Nmda] Receptor Subunit Zeta 1	1y20	A	1pbq	A
Nuclear Factor Of Activated T-Cells, Cytoplasmic 2	1owr	Q	1owr	M
Non-Toxin Haemagglutinin Ha34	1ybi	B	1ybi	A
Potassium Channel	2wln	A	2wlk	A
Nuclear Factor Of Activated T Cells 5	1imh	C	1imh	D
Mannose-Specific Adhesin Fimh	1qun	J	1qun	H
Pantothenate Synthetase	3ag5	A	3ag6	A
Hla-Cw3 (Heavy Chain)	1efx	A	1qqd	A
H-2 Class I Histocompatibility Antigen D-B Alpha Chain	2cii	A	1ffo	D
Diaminopimelate Epimerase	2gke	A	1gqz	A
Mhc Class I H-2dd Heavy Chain	1qo3	A	1ddh	A
Glutamate Receptor Ionotropic, Nmda 2A	4nf5	B	3oen	A
Nad-Dependent Deacetylase Sirtuin-3, Mitochondrial	3gls	F	3glr	A
Zinc-Alpha-2-Glycoprotein	1t7z	A	1zag	A
Glycine Betaine/carnitine/choline-Binding Protein	3ppo	A	3ppn	A
D-Ribose-Binding Protein	1ba2	A	1urp	C
Osmoprotection Protein (Prox)	1sw4	A	1sw5	C
Udp-2,3-Diacylglucosamine Pyrophosphatase Lpxi	4ggm	X	4j6e	A

Interferon-Induced, Double-Stranded Rna- Activated Protein Kinase	2a1a	B	2a19	C
Coat Protein	1opo	C	1opo	A
N-Methyl-D-Aspartate Receptor Subunit 1	1pbq	B	1pb7	A
Pectocin M2	4n58	A	4n59	A
Spac19a8.12 Protein	2qkm	B	2qkm	D
Gamma-Snap	2ifu	B	2ifu	A
Shikimate Dehydrogenase	2hk8	F	2hk9	C
Phosphate-Binding Protein Psts 1	4exl	A	4lat	A
Probable Translation Initiation Factor 2 Alpha Subunit	1yz6	A	1yz7	A
Glutamate Receptor Subunit 2	1ftj	B	1fto	A
Uroporphyrinogen-III Synthase	1jr2	A	1jr2	B
Acetylglutamate Kinase	1oha	A	2wxb	A
Glutamate Receptor, Ionotropic Kainate 2	1s50	A	1fto	A
Glutamate Receptor 3	3dln	A	1fto	A
Glutamate Receptor 4,Glutamate Receptor	3en3	A	1fto	A
Glutamate Receptor, Ionotropic Kainate 1	2f34	B	1ycj	B
Betaine Abc Transporter Permease And Substrate Binding Protein	3l6g	A	3l6h	A
3-Deoxy-Manno-Octulosonate Cytidylyltransferase	1vic	A	1vh3	B
Heat Shock Protein Hsp 90-Beta	3pry	A	1usu	A
Glutamate Receptor, Ionotropic Kainate 3	4e0w	A	1fto	A
Nopaline-Binding Periplasmic Protein	4pow	A	4p0i	A
Angiostatin	1ki0	A	2doh	X
Endonuclease VIII	1k3x	A	1q3b	A
Major Surface Antigen P30	1kzq	A	1ynt	G
DNA Topoisomerase I	1cyy	B	1cyy	A
Alpha-1 Catenin	1h6g	A	1l7c	C
Nadh Pyrophosphatase	1vk6	A	2gb5	A
Mg2+ Transporter Mgte	2yvy	A	2yvz	A
Replication Protein A 70 Kda Dna-Binding Subunit	1fgu	B	1fgu	A
Dihydrodipicolinate Reductase	1yl7	C	1p9l	A
Hypothetical Protein	2i76	A	2i76	B
Nad-Dependent Deacetylase	2h2i	A	2h59	B
Dihydrodipicolinate Reductase	3qy9	B	3qy9	D
Putative Abc Transporter, Periplasmic Binding Protein, Amino Acid	2yln	A	3zsf	A
Chlorophenol Reduction Gene K	2h6b	A	2h6b	B
Lysine, Arginine, Ornithine-Binding Protein	2lao	A	1lst	A
Nuclease	2gki	B	2gki	A
Glutamate Receptor Delta-2 Subunit	2v3t	A	2v3u	A
Windbeutel Protein	2c1y	A	2c0e	A
Dna-Directed RNA Polymerase Alpha Chain	1ynj	A	1ynn	B
Dna-Directed RNA Polymerase Alpha Chain	2a6h	A	1iw7	L
Fab 17B Heavy Chain	1rz8	B	2i60	R
Igg1 Antibody 58.2 (Heavy Chain)	1f58	H	3f58	H
Fab 2219, Heavy Chain	2b0s	H	2b1h	H

Abc-Type Transporter, Periplasmic Subunit Family 3	4psh	A	4prs	A
Fab-Ysd1 Heavy Chain	1za3	H	1za3	B
Glutamine Binding Protein	1wdn	A	1ggg	B
Hyb3 Heavy Chain	1w72	H	1dfb	H
Calcium-Gated Potassium Channel Mthk	2fy8	C	2fy8	H
Chimera Of Fab2c4: "Humanized" Murine Monoclonal Antibody	1l7i	H	1s78	F
Fab Fragment From Human Immunoglobulin Igg1 (Lambda, Hil)	8fab	D	8fab	B
Igg1 Fab Fragment	1igc	H	2aab	H
Catalytic Elimination Antibody 13G5 Heavy Chain	2gjz	B	2gk0	H
Chimeric Germline Precursor Of Oxy-Cope Catalytic Antibody Az-28 (Heavy Chain)	1d5i	H	1d5b	B
Fab Fragment, Heavy Chain	2h2s	E	2htl	C
Igg1 Fab Fragment (Hc19)	1gig	H	2vir	B
Antibody Light Chain	1jgu	L	1baf	L
Fab Fragment, Antibody A5b7	1ad0	B	1rmf	H
Heavy Chain Of A Vegf Binding Antibody	1n8z	B	2fjg	B
Igg Heavy Chain	2dd8	H	1rzi	F
Fab Fragment Of 8F5 Antibody Against Human Rhinovirus 3 Serotype 2 4	1bbd	L	1hin	L
Immunoglobulin Igg1 Heavy Chain	2fl5	D	2fl5	B
Natrin 1	1xta	A	2giz	A
Syk Kinase	1a81	G	1a81	E
Antibody Germline Precursor To 28B4	1fl6	B	1fl5	H
Fab (Bv04-01)	1nbv	H	1cbv	H
K42-41l Fab Light Chain	1mju	L	1uz8	A
28B4 Fab	1kem	H	1q9l	B
82D6a3 Igg	2adf	H	2brr	H
Immunoglobulin Heavy Chain	2ai0	K	2ai0	I
Pc283 Immunoglobulin	1kcr	H	1kcu	H
Anti-Idiotypic Monoclonal Antibody (Light Chain)	2aab	L	1iqw	L
Igg 5C8	15c8	H	1fgn	H
Antibody M41	1gpo	L	1keg	L
Fab Heavy Chain	1xf3	H	1i8m	B
Immunoglobulin Gamma-1 Heavy Chain Constant Region	1e4k	B	2iwg	A
Igg1 Antibody 58.2 (Light Chain)	1f58	L	3f58	L
Immunoglobulin G1 (Igg1)	2mcg	1	2mcg	2
Loc - Lambda 1 Type Light-Chain Dimer	3bjl	B	1bjm	A
Mature Metal Chelatase Catalytic Antibody, Heavy Chain	1ngy	B	1n7m	L
Dihydrodipicolinate Reductase	1vm6	C	1vm6	B
Fab 2219, Light Chain	2b0s	L	2b1h	L
Igg2a Fab Fragment (50.1)	1ggi	M	1ai1	L
Igg2a Fab Fragment (50.1)	1ggi	H	1ggb	H
Igm-Kappa Cold Agglutinin (Light Chain)	1dn0	C	1rhh	A
Immunoglobulin Lambda Light Chain	1jvk	B	1jvk	A

Monoclonal Antibody 2D12.5, Igg1 Gamma Heavy Chain	1gig	L	1q0x	L
Antibody Light Chain 11K2	2bdn	L	1osp	L
Immunoglobulin 48G7 Germline Fab	1gaf	L	1gpo	L
Fab 17B Light Chain	1rz8	A	2ny1	C
Fab Fragment Of Murine Monoclonal Antibody An02 Complex 3 With Its Hapten (2,2,6,6-Tetramethyl-1-Piperidinyloxy- 4 Dinitrophenyl)	1baf	L	1cz8	L
Fab, Heavy Chain	1mhh	D	1xcq	F
33H1 Fab Light Chain	1ors	A	1fig	L
Humanized Antibody Hfe7a, Light Chain	1it9	L	2gcy	A
Igg2a Intact Antibody - Mab231	1igt	C	1igt	A
Igg Antibody (Light Chain)	1emt	L	2a6i	A
Igg2b (Kappa)	1cgs	H	2cgr	H
Monoclonal Anti-Estradiol 10G6d6 Immunoglobulin Gamma-1 Chain	1jn6	B	1jnh	B
Pc287 Immunoglobulin	1kcu	L	1fsk	K
Antibody Light Chain Fab	1i8m	A	1qbm	L
Erythropoietin Receptor	1eer	B	1ern	B
17E8	1eap	A	1a0q	L
Fab E51 Light Chain	1rzf	L	1q1j	M
Fab Fragment, Light Chain	1mhp	L	1ad0	C
Germline Metal Chelatase Catalytic Antibody, Chain H	1n7m	H	1ngy	A
Humanized Antibody D3h44	1pg7	H	1jps	H
IgA Fab Fragment (J539)	2fbj	L	2fd6	L
Immunoglobulin	1ce1	L	1t04	C
Lambda III Bence Jones Protein Cle	1lil	A	1lil	B
Cetuximab Fab Light Chain	1yy9	C	1yy8	A
Chimeric Germline Precursor Of Oxy-Cope Catalytic Antibody Az-28 (Light Chain)	1d6v	L	1axs	L
Heavy Chain Vh+ch1 Anti-Lysozyme Antibody F10.6.6	1p2c	E	1mlc	B
Hyb3 Light Chain	1w72	M	1adq	L
Fibroblast Growth Factor Receptor 2	1e0o	D	1djs	A
Septum Site-Determining Protein Minc	1hf2	C	1hf2	A
Vascular Cell Adhesion Molecule-1	1vca	A	1ij9	A
P58-Cl42 Kir	1nkr	A	2dl2	A
Arno	1pbv	A	1r8s	E
N2b-Titin Isoform	2f8v	C	2a38	C
Transcriptional Regulator, Tetr Family	1zkg	A	1z77	A
Intercellular Adhesion Molecule-1	1ic1	B	1ic1	A
Hypothetical Transcriptional Regulator In Qaca 5''Region	1jt6	A	1jt0	D
Muscle-Specific Kinase Receptor	2iep	B	2iep	A
Calcium And Integrin Binding 1 (Calmyrin)	1y1a	A	1xo5	A
Transcriptional Regulator	3vok	A	3vp5	A
Tenascin	1qr4	B	1qr4	A

Fatty Acid-Binding Protein, Epidermal	4azr	B	1b56	A
