# Supplementary material for: Investigation of sequence features of hinge-bending regions in proteins with domain movements using kernel logistic regression
Source: BMC Bioinformatics. 2020 Apr 9;21:137. doi: 10.1186/s12859-020-3464-3 (PMC7147021; doi:10.1186/s12859-020-3464-3)
Supplement: Supplementary file 2 — Additional file 2: Table S1. Table giving matrix of p-values for the pairwise comparisons of the AUROC for the linear, quadratic, cubic and RBF models for Group1_90% dataset. [file 12859_2020_3464_MOESM2_ESM.pdf]

Additional Table 1 Matrix of p-values for comparisons of the AUROCs for models with window length 99 using the Group1_90% dataset.

	**Linear**	**Quadratic**	**Cubic**	**RBF**
**Linear**	-	1.17×10^{-5}	0.0013	2.22×10^{-5}
**Quadratic**	-	-	0.0811	0.0091
**Cubic**	-	-	-	0.8983
**RBF**	-	-	-	-
