# Supplementary material for: Investigation of sequence features of hinge-bending regions in proteins with domain movements using kernel logistic regression
Source: BMC Bioinformatics. 2020 Apr 9;21:137. doi: 10.1186/s12859-020-3464-3 (PMC7147021; doi:10.1186/s12859-020-3464-3)
Supplement: Supplementary file 3 — Additional file 3: Table S2. Table giving matrix of p-values for the pairwise comparisons of the AUROC for the linear, quadratic and cubic models for Group2_90% dataset. [file 12859_2020_3464_MOESM3_ESM.pdf]

Additional Table 2 Matrix of p-values for comparisons of the AUROCs for models with window length 101 using the Group2_90% dataset.

	**Linear**	**Quadratic**	**Cubic**
**Linear**	-	4.44×10^{-16}	0.0111
**Quadratic**	-	-	3.61×10^{-8}
**Cubic**	-	-	-
