# Supplementary material for: Investigation of sequence features of hinge-bending regions in proteins with domain movements using kernel logistic regression
Source: BMC Bioinformatics. 2020 Apr 9;21:137. doi: 10.1186/s12859-020-3464-3 (PMC7147021; doi:10.1186/s12859-020-3464-3)
Supplement: Supplementary file 4 — Additional file 4: Table S3. Table for comparison of AUROCs for window lengths 81 and 87. [file 12859_2020_3464_MOESM4_ESM.pdf]

Additional Table 3 Comparison of AUROC's for window lengths 81 and 87.

	Group1_90%		Goup1_40%		Group1_20%	
**Model:**	**Linear**	**Quadratic**	**Linear**	**Quadratic**	**Linear**	**Quadratic**
**Window 81:**	0.685	0.749	0.579	0.613	0.552	0.569
**Window 87:**	0.687	0.749	-	-	0.553	0.569
