# Supplementary material for: Investigation of sequence features of hinge-bending regions in proteins with domain movements using kernel logistic regression
Source: BMC Bioinformatics. 2020 Apr 9;21:137. doi: 10.1186/s12859-020-3464-3 (PMC7147021; doi:10.1186/s12859-020-3464-3)
Supplement: Supplementary file 5 — Additional file 5: Figure S1. HingeIndex values for amino acids evaluated from Group2_90% dataset. [file 12859_2020_3464_MOESM5_ESM.pdf]

Additional Figure 1

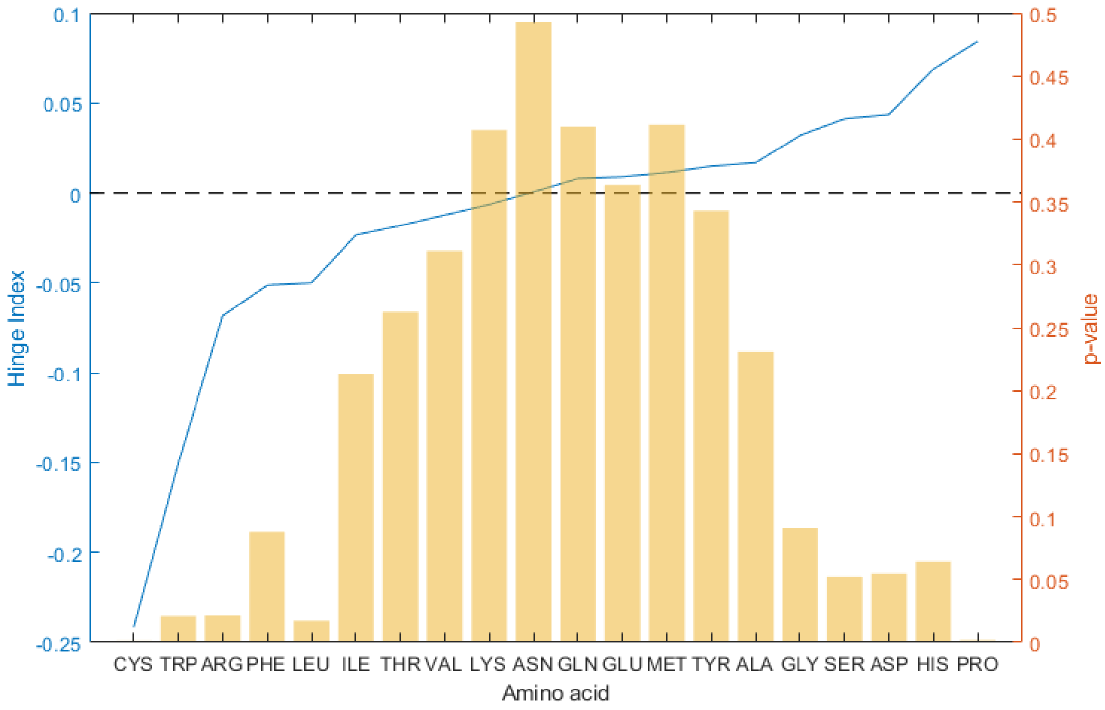

Propensities (Hinge Index) of amino acids for hinge-bending regions and p-values. The propensity (HI value) of each amino acid in the hinge-bending regions of Group 2 at 90% filtering. See main text for details.
