# Supplementary material for: Investigation of sequence features of hinge-bending regions in proteins with domain movements using kernel logistic regression
Source: BMC Bioinformatics. 2020 Apr 9;21:137. doi: 10.1186/s12859-020-3464-3 (PMC7147021; doi:10.1186/s12859-020-3464-3)
Supplement: Supplementary file 6 — Additional file 6: Figure S2. (A) ROC curves for the quadratic model with window length 81 on Group1_90% with various proportions of positive to negative training examples. (B) Plots of the AUROC against proportion of positive to negative training examples for different window lengths. [file 12859_2020_3464_MOESM6_ESM.pdf]

Additional Figure 2

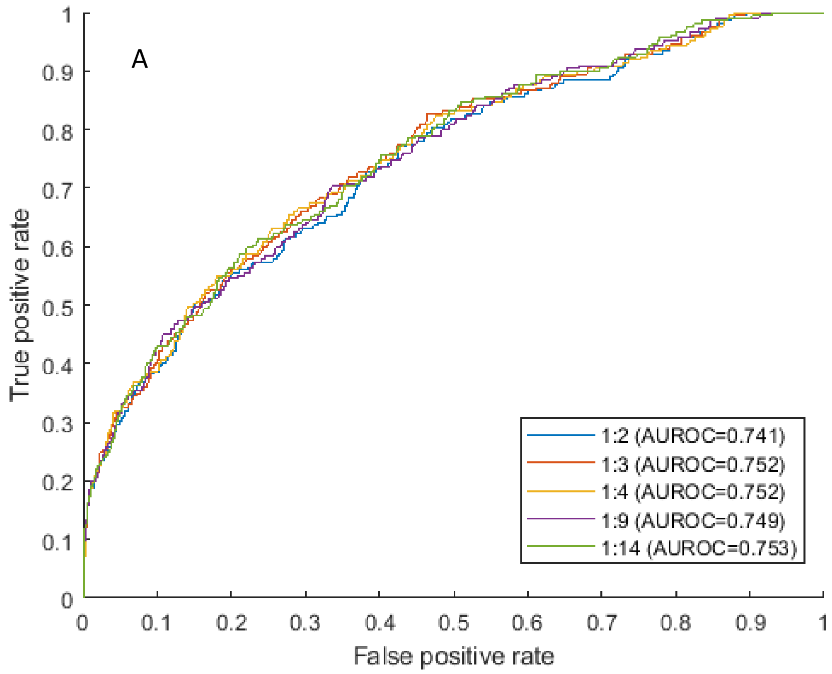

(A) ROC curves for the quadratic KLR model using Group1_90% data with window length 81. Each curve is for a different proportion of positive to negative training examples. As can be seen from the AUROC values inset there is little variation in the AUROC for different proportions of positive to negative training examples.

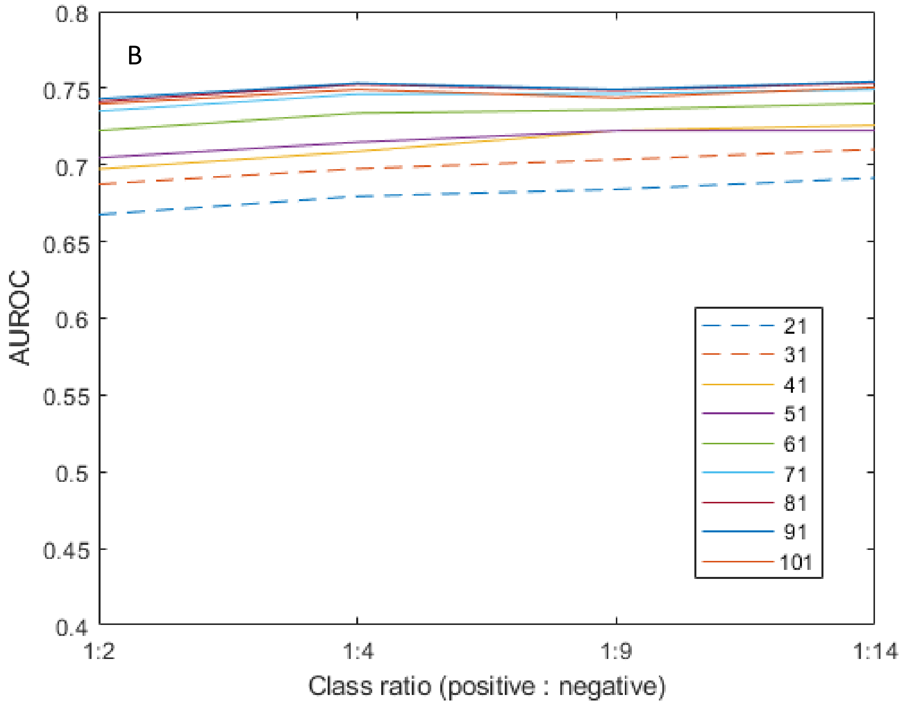

(B) Plots of the AUROC against proportion of positive to negative training examples for quadratic KLR model using Group1_90% data. Each curve shows the result for a different window length. This confirms that there is little variation of the AUROC against variation in this proportion. Note the AUROC axis starts at 0.4.
