# Supplementary material for: Investigation of sequence features of hinge-bending regions in proteins with domain movements using kernel logistic regression
Source: BMC Bioinformatics. 2020 Apr 9;21:137. doi: 10.1186/s12859-020-3464-3 (PMC7147021; doi:10.1186/s12859-020-3464-3)
Supplement: Supplementary file 7 — Additional file 7: Figure S3. Precision-Recall curve for Group1_90%. [file 12859_2020_3464_MOESM7_ESM.pdf]

Additional Figure 3

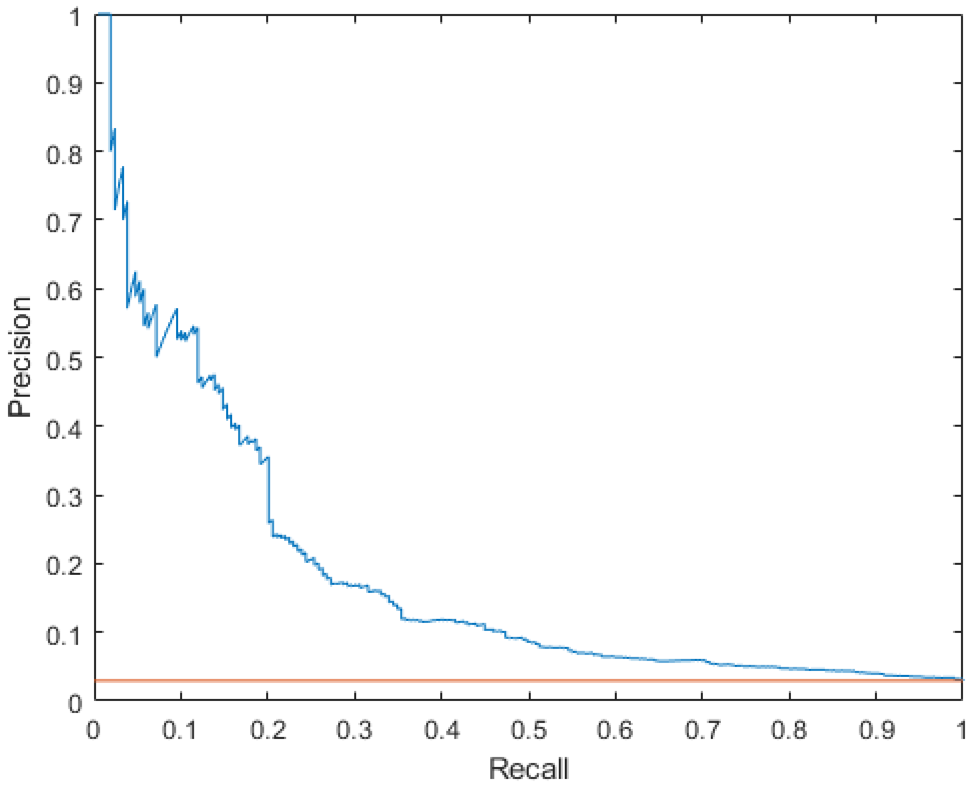

Precision-Recall curve (blue) for Group1_90%, quadratic KLR model with window length 81. The red line shows the proportion of hinge residues to all residues in the test set which would be the area under the Precision-Recall curve (AUPRC) for a random classifier. The AUPRC is 0.1785 and the red line is at 0.0294.
