# Supplementary material for: Investigation of sequence features of hinge-bending regions in proteins with domain movements using kernel logistic regression
Source: BMC Bioinformatics. 2020 Apr 9;21:137. doi: 10.1186/s12859-020-3464-3 (PMC7147021; doi:10.1186/s12859-020-3464-3)
Supplement: Supplementary file 8 — Additional file 8: Figure S4. Area under Precision-Recall curves for different KLR models at different window lengths for Group1_90% dataset. [file 12859_2020_3464_MOESM8_ESM.pdf]

Additional Figure 4

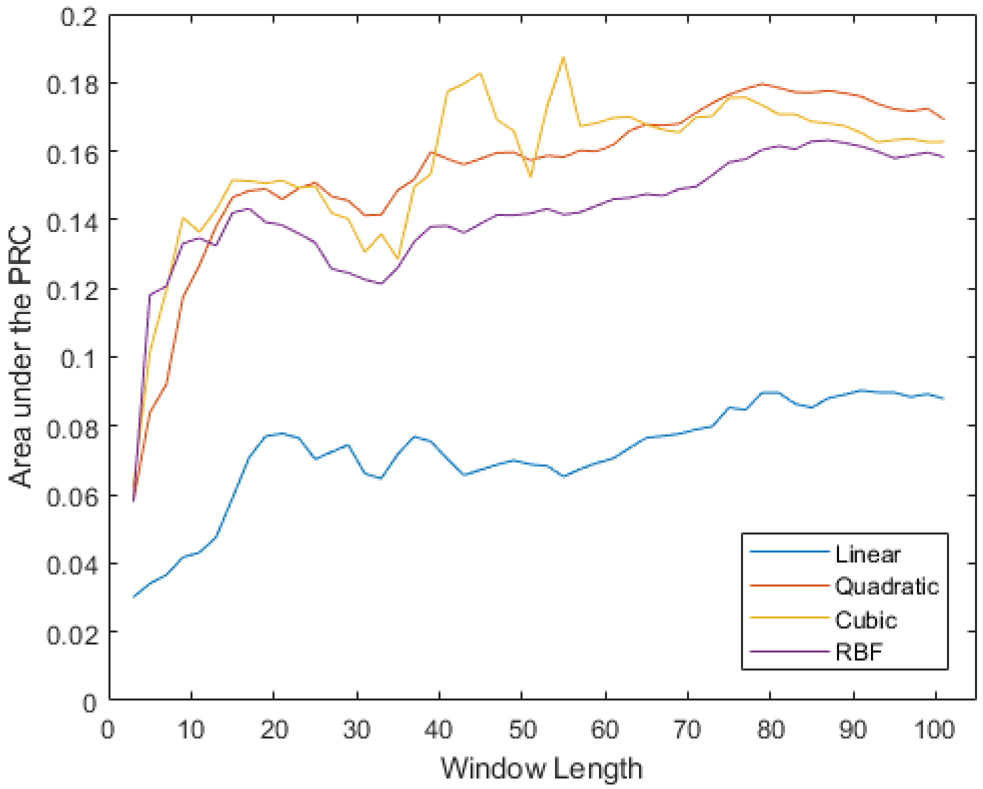

AUPRC's plotted against window length with the Group1_90% dataset for the four different KLR models investigated.
