# Supplementary material for: Investigation of sequence features of hinge-bending regions in proteins with domain movements using kernel logistic regression
Source: BMC Bioinformatics. 2020 Apr 9;21:137. doi: 10.1186/s12859-020-3464-3 (PMC7147021; doi:10.1186/s12859-020-3464-3)
Supplement: Supplementary file 9 — Additional file 9: Figure S5. p-values at different window lengths for the Group1_40% dataset determined by doing a paired t-test of the AUROC between the linear and quadratic KLR models. [file 12859_2020_3464_MOESM9_ESM.pdf]

Additional Figure 5

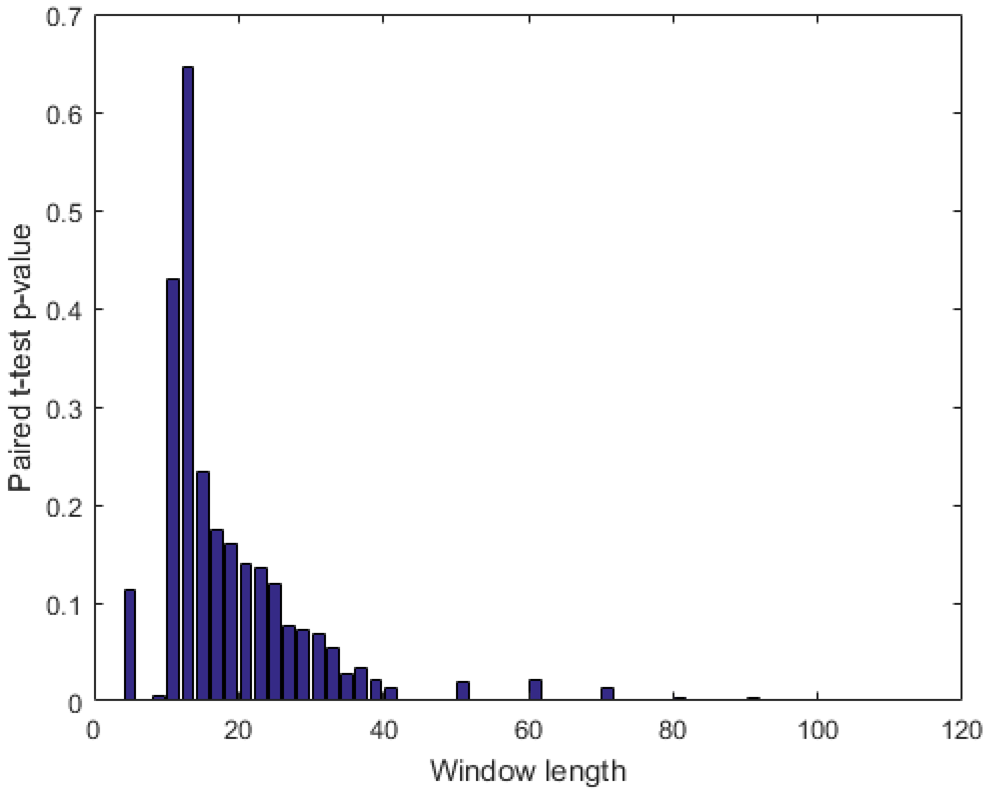

p-values at different window lengths for the Group1_40% dataset determined by doing a paired t-test of the AUROC on each fold for the linear and quadratic KLR models.
